# A 3′-end structure in RNA2 of a crinivirus is essential for viral RNA synthesis and contributes to replication-associated translation activity

**DOI:** 10.1038/srep34482

**Published:** 2016-10-03

**Authors:** Chawin Mongkolsiriwattana, Jaclyn S. Zhou, James C. K. Ng

**Affiliations:** 1 Department of Plant Pathology and Microbiology, University of California, Riverside, Riverside, California, USA

## Abstract

The terminal ends in the genome of RNA viruses contain features that regulate viral replication and/or translation. We have identified a Y-shaped structure (YSS) in the 3′ terminal regions of the bipartite genome of *Lettuce chlorosis virus* (LCV), a member in the genus *Crinivirus* (family *Closteroviridae*). The YSS is the first in this family of viruses to be determined using Selective 2′-Hydroxyl Acylation Analyzed by Primer Extension (SHAPE). Using luciferase constructs/replicons, *in vivo* and *in vitro* assays showed that the 5′ and YSS-containing 3′ terminal regions of LCV RNA1 supported translation activity. In contrast, similar regions from LCV RNA2, including those upstream of the YSS, did not. LCV RNA2 mutants with nucleotide deletions or replacements that affected the YSS were replication deficient. In addition, the YSS of LCV RNA1 and RNA2 were interchangeable without affecting viral RNA synthesis. Translation and significant replication were observed for specific LCV RNA2 replicons only in the presence of LCV RNA1, but both processes were impaired when the YSS and/or its upstream region were incomplete or altered. These results are evidence that the YSS is essential to the viral replication machinery, and contributes to replication enhancement and replication-associated translation activity in the RNA2 replicons.

Members of the genus *Crinivirus* (family *Closteroviridae*) are whitefly-transmitted, emerging plant viruses and affiliates of the alphavirus-like supergroup of single-stranded (ss), positive (+)-sense RNA viruses with large (15.3–17.6 kb) and complex genomes^1^,^2^. Cloned infectious cDNAs of the bipartite genomic (g)RNAs of the crinivirus *Lettuce chlorosis virus* (LCV) have been developed, allowing comparative inferences with *Lettuce infectious yellows virus* (LIYV; the type species of *Crinivirus*) and viruses in the related genus *Closterovirus*^3^,^4^,^5^,^6^. As with other criniviruses, the gRNAs of LCV are capped at the 5′ end and not polyadenylated at the 3′ end^7^,^8^. Cap-dependent translation of (+)-strand LCV RNA1 results in the production of the viral replicase consisting of the open reading frame (ORF) 1a encoded papain-like leader protease (P-Pro), methyltransferase (MTR) and helicase (HEL), and the ORF 1b encoded RNA-dependent-RNA polymerase (RdRp) (Fig. 1a)^8^,^9^. Consequently, LCV RNA1 can replicate on its own^5^,^10^, resulting in the production of complementary minus (−)-strand and (+)-strand RNAs. The (−)-RNA serves as a template for the synthesis of subgenomic (sg)RNAs from which P8, a putative protein of unknown function, and P23, a viral suppressor of RNA silencing^11^ (Fig. 1a), are translated^12^. LCV RNA2 replicates using the RNA1-encoded replicase supplied in *trans*^5^,^10^; none of its 10 ORFs (Fig. 1a) are known to be involved in replication^8^,^12^. The first ORF (P5.6), whose function is unknown, by virtue of its position in RNA2, may be expressed by cap-dependent translation of the (+)-RNA but this has yet to be determined experimentally. Expression of the 3′ proximal ORFs is via a nested set of 3′ co-terminal sgRNAs made using (−)-strand RNA2 as the template^12^.

The 3′ terminus in the genome of RNA viruses contain secondary structures that, on their own or in combination with others through RNA-RNA interactions, are associated with many important functions, including the initiation and regulation of (−)-RNA synthesis, translation regulation, and virion encapsidation^13^,^14^,^15^. The 3′ non-coding region (NCR) of the LCV gRNAs contains a highly structured heteropolymeric sequence that is predicted to be free of pseudoknots^12^. The 3′ NCR of RNA1 is 226-nucleotide (nt) long and shares 74% sequence identity with its 226-nt counterpart in RNA2. In this 226-nt region of RNA2, the first 128 nts are part of the ORF encoding P4.8, while the downstream 98 nts constitute the 3′ NCR of the RNA. The sequence identity between the 98-nt region (from here on referred to as the “98-nt”) of RNAs 1 and 2 is 81%^12^ (Fig. 1b). Neither structures nor functions of the 3′ NCRs in the LCV genome or the genomes of any criniviruses have as yet been investigated.

In this study, using Selective 2′-Hydroxyl Acylation Analyzed by Primer Extension (SHAPE)^16^,^17^,^18^,^19^ analysis of full-length LCV RNAs 1 and 2, we have identified a Y-shaped structure (YSS) consisting of two stem-loops, SL1 and SL2, and a closing stem, S3, in the 3′ terminal regions of both RNAs, and have investigated its role in translation by performing *in vivo* and *in vitro* translation assays using constructs containing the *firefly* luciferase (F-Luc) coding sequence flanked with the 5′ NCR and the YSS-containing 3′ NCR of LCV RNAs 1 or 2. We have also investigated the role of the YSS in supporting viral RNA synthesis by assessing the relative amounts of progeny RNA produced when 3′ NCR/YSS mutants of LCV RNA2 are co-inoculated with WT LCV RNA1 to tobacco protoplasts. The implications of our findings for conceptualizing the role of the YSS in viral RNA synthesis and translation for LCV and other criniviruses are discussed.

## Results

### SHAPE analysis of the 3′-terminal regions of LCV RNAs 1 and 2

Substantial studies have demonstrated that secondary structures of (+)-RNA viruses participate in various regulatory functions^15^,^20^,^21^,^22^,^23^,^24^,^25^,^26^. To identify potential regulatory structures in the 3′ region of the LCV RNA genome, we used SHAPE to analyze full-length LCV gRNAs 1 and 2 (see Methods). This resulted in the identification of a similar but not identical Y-shape structure (YSS) in both RNAs (Fig. 2a,b). The YSS (RNAs 1 and 2 nt position 8483–8576 and 8450–8541, respectively) consists of two apical SLs, named SL1 and SL2, and a basal stem, S3, that forms the closing stem (of the YSS) (Fig. 2a,b). SL1 spans nt position 8530–8568 and 8495–8533 of RNA1 and RNA2, respectively. SL2 of RNA1 (nt position 8492–8529 of RNA1) and SL2 of RNA2 (nt position 8460–8491 of RNA2) are slightly different from each other. SL2 of LCV RNAs 1 and 2 both consist of 9 base pairs (bp) in the lower stem, 4 nts in the internal loop, 3 bp in the upper stem and 4 nts in the loop (Fig. 2a,b). However, SL2 of LCV RNA1 has a mismatch and two additional base-pairs in the lower stem (Fig. 2a,b). Furthermore, SL1 and SL2 of LCV RNA1 are not separated by any nucleotide, while both SLs of LCV RNA2 are separated by 3 nts, of which two are moderately reactive to BzCN modification. Both SLs of RNAs 1 and 2 are united at the base of the Y-configuration by an A-U base-pair (RNA1 nt position 8490 and 8569; RNA2 nt position 8457 and 8534). The union at the base continues on through 7 bp to form S3 (Fig. 2a,b). Most of the unpaired nts in the SLs were moderately-highly reactive to BzCN modification (see methods). With the exception of 2 nts in the loop of SL1 in LCV RNA1, all nts in the loop of both SLs in LCV RNAs 1 and 2 were moderately-highly reactive to BzCN modification (Fig. 2a,b), suggesting that these nts were not constrained by interactions with other parts of the RNA. In RNA2, nts that form the the A-U base-pair (position 8474 and 8479) and the U-A base-pair (position 8473 and 8480) were moderately reactive to BzCN modification, whereas in RNA1, nts that form similar base-pairs (A-U at nt position 8509 and 8514, and U-A at nt position 8508 and 8515) were unreactive to BzCN modification. This suggests that the loop in SL2 of RNA2 may be larger than that in SL2 of RNA1. The S3s of RNAs 1 and 2 appear very similar, with only one nt (A) at position 8490 in RNA1 being moderately reactive to BzCN (Fig. 2a,b).

### Luciferase assays to determine the role of the YSS in translation

**Figure 1:**
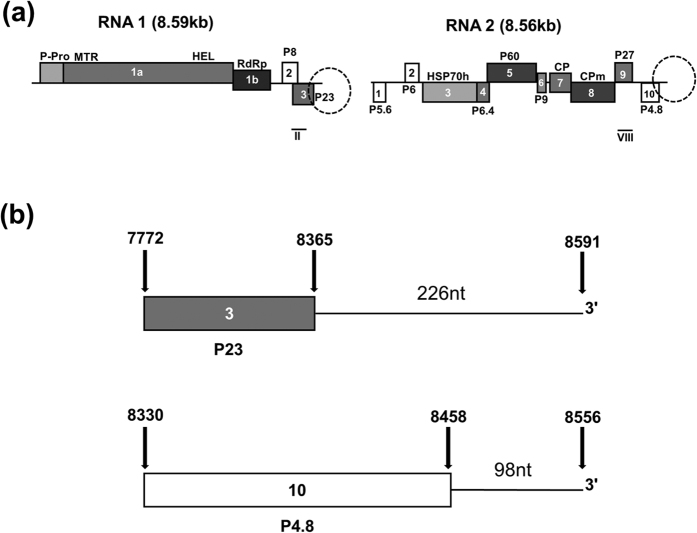
LCV genome organization and layout of the 3′ non-coding region. (**a**) A schematic representation of the LCV genome. Open reading frames (ORFs; 1a – 3 and 1–10 in LCV RNAs 1 and 2, respectively) encoding the following predicted viral proteins are as indicated: P-Pro, papain-like protease; MTR, methyltransferase; HEL, RNA helicase; RdRp, RNA-dependent RNA polymerase; HSP70h, heat shock protein 70 homolog; CP, major coat protein; CPm, minor coat protein; and proteins that are named according to their relative molecular masses (indicated by numbers preceded by “P”): P8, P23, P5.6, P6, P6.4, P60, P9, P27, and P4.8. Black bars below the genome map represent DIG-labeled riboprobes (II and VIII) complementary to the corresponding locations in the genomic RNAs. (**b**) Enlargement of the areas indicated by the dashed-circles (in Fig. 1a) representing the 3′ terminal region of LCV RNAs 1 (top) and 2 (bottom). Numbers above the arrows indicate the nucleotide positions on both RNAs.

To determine if the YSS contributes to viral translation, we performed *in vivo* translation assays using a series of F-Luc reporter constructs (see Methods), and translation efficiency was determined by the ratio of the F-Luc/R-Luc measurements (with R-Luc serving as an internal control). In constructs LUC-TMV, LUC-R1 and LUC-R2A, the F-Luc gene was flanked by the 5′ and 3′ NCRs of *Tobacco mosaic virus* (TMV) RNA, LCV RNA1 and LCV RNA2, respectively (Fig. 3a). Relative F-Luc activity was observed for LUC-TMV (Fig. 3b), demonstrating the TMV NCRs’ role in translation. The relative F-Luc activity of LUC-R1 was lower than that of LUC-TMV but significantly higher than that of LUC-R2A, which in turn was not significantly different from that of the water control inoculations (Fig. 3b). These results suggest that 5′ and 3′ NCRs of LCV RNA1, but not those of LCV RNA2, support translation activity. We also assessed the luciferase activity of LUC-R2A(−) (Fig. 3a), a modified LUC-R2A in which the 3′ NCR (98-nt) of LCV RNA2 was substituted with 98 non-viral nts (taken from the GFP coding sequence). The relative F-Luc activities of LUC-R2A(−) and LUC-R2A were comparable (Fig. 3b), and both were not significantly different from that of the water control. These results suggest that the 5′ and 3′ NCRs of LCV RNA2 (the latter covering all but 9 nts of the YSS) do not contribute to translation of LCV RNA2. We next broaden the area of analysis by using LUC-R2B and LUC-R2C, both of which contained the complete 5′ and 3′ NCRs of LCV RNA2 in addition to 9 nts (in LUC-R2B) and 302 nts (in LUC-R2C) taken from the region immediately upstream of the 3′ NCR. These extra nts enabled complete coverage of the YSS (LUC-R2B) as well as the YSS plus the entire P4.8 coding sequence and the intergenic region between the P4.8 and P27 ORFs (LUC-R2C) (Fig. 3a). However, the relative F-Luc activities of LUC-R2B and LUC-R2C were comparably as low as that of LUC-R2A and both were not significantly different from that of the water control (Fig. 3b). Studies of Sindbis virus and *Saguaro cactus virus* (SCV) have shown that incorporating into luciferase constructs different number of additional nts from the 5′ proximal ORF of Sindbis virus sgRNA and SCV gRNA, respectively^27^,^28^, can result in different levels of luciferase activities. This prompted us to extend the 5′ NCR sequence in LUC-R2A and LUC-R2C by adding to it 99 nts from the proximal 5′ end of the P5.6 ORF (Fig. 1a), yielding LUC-R2D and LUC-R2E, respectively (Fig. 3a). Still, the relative F-Luc activity of LUC-R2D and LUC-R2E were not significantly different from that of LUC-R2A and LUC-R2C, respectively (Fig. 3b). We next deleted SL1 or SL2 in LUC-R2C but the resulting constructs, LUC-R2CΔSL1 and LUC-R2CΔSL2 (Fig. 3a), showed no significant changes in relative luciferase activity compared to that of LUC-R2C (Fig. 3b), and to each other.

Translation activity for all of the above F-Luc constructs was also determined using *in vitro* assays and the results were consistent with those of the *in vivo* assays (Supplementary Note S1; Supplementary Fig. S1). Collectively, these results demonstrate that the 5′ and 3′ NCRs of LCV RNA2 (including an extensive 400-nts 3′ region that encompasses the YSS) do not contribute to translation of the RNA of the F-Luc constructs. Translation activity was observed for specific F-Luc constructs only when the transcripts were co-inoculated with LCV RNA1 (see later).

### Mapping regions in the 3′ end of LCV RNA2 involved in viral RNA synthesis

Results from the preceding section raise the possibility that LCV gRNA2 does not itself possess messenger activity, and beg the question of what role the YSS-containing 3′ NCR might play in the LCV infection process. Here, we used a series of LCV RNA2 3′ NCR deletion mutants to investigate whether it is involved in viral RNA synthesis. Given that the 3′ NCR of LCV RNA1 supports translation, it is not an ideal template for making these mutations since they could affect translation, viral RNA synthesis, or both, thus making negative results difficult to interpret.

Capped *in vitro* transcripts of each RNA2 mutant were co-inoculated with that of WT LCV RNA1 into tobacco protoplasts, and the effects of the deletions on viral RNA synthesis were determined. (−)-RNA and (+)-RNA synthesis of 3′Δ4 were comparable to that of the WT (Fig. 4a,b). To determine whether additional nts located upstream of those deleted in 3′Δ4 (but downstream of those that form the YSS) are involved in viral RNA synthesis, we constructed and tested 3′Δ11 (Fig. 4a). This deletion was predicted by *mfold* to not disrupt the YSS. Viral RNA accumulation of 3′Δ11 was detected at a lower level, with (−)-RNA accumulating at approx. 39% compared to that of the WT (Fig. 4c). Additional mutants, 3′Δ24, 3′Δ38 and 3′Δ48, were further constructed and tested. All 8 nts that form the right basal stem of the YSS were deleted in these three mutants. In addition, 1, 15 and 25 nts that form SL1 were deleted in 3′Δ24, 3′Δ38 and 3′Δ48, respectively (Fig. 4a). We also constructed mutant 5′Δ50 (with a deletion of 50 nts from the proximal 5′ end of the 98-nt, which covers all the nts that form SL2 and 14 nts that form SL1) (Fig. 4a). Northern analysis showed that 3′Δ24 accumulated viral RNA at a lower level, with (−)-RNA accumulating at approx. 12.7% compared to the WT (Fig. 4d), while 3′Δ38, 3′Δ48, and 5′Δ50 failed to accumulate viral RNA (Fig. 4e,f). To ensure that all engineered mutations were retained in the (−)-RNA progenies of the viable LCV RNA2 mutants (3′Δ4, 3′Δ11 and 3′Δ24), we performed 5′ RACE analysis using the total RNA of the inoculated protoplasts (harvested at 96 hpi). In all cases, the engineered deletions were retained in the (−)-RNA progenies.

### Mutations targeting SL1 and SL2 in the YSS of LCV RNA2

**Figure 2:**
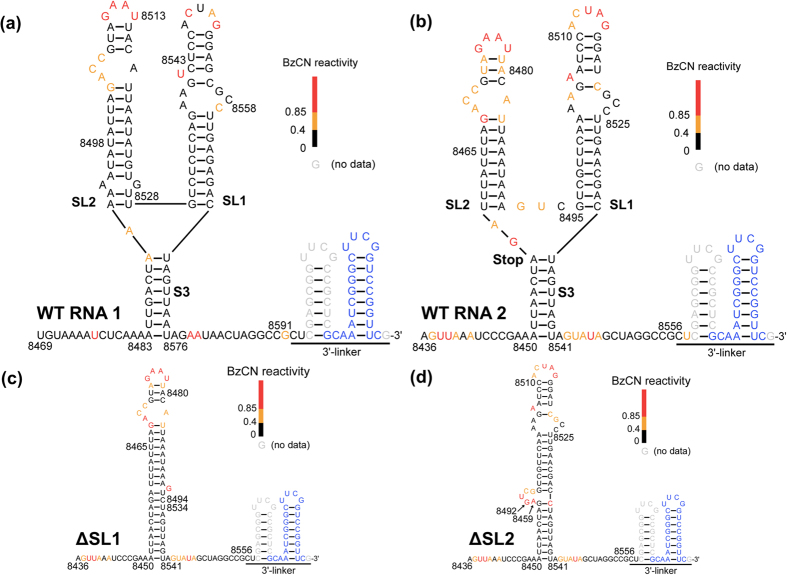
SHAPE analysis of LCV RNAs 1 and 2. Secondary structure models of the 3′ terminal region in: (**a**) wild-type (WT) LCV RNA1, (**b**) WT LCV RNA2, (**c**) ΔSL1 (LCV RNA2 with stem-loop [SL]1 deleted), and (**d**) ΔSL2 (LCV RNA2 with SL2 deleted). RNA secondary structures were generated from the *RNAstructure* software, with benzoyl cyanide (BzCN) reactivity incorporated as pseudo-free energy constraints. Each colored nucleotide corresponds to the level of BzCN reactivity for that particular nucleotide, with black, yellow, and red representing unreactive (0–0.4), moderately reactive (0.4–0.85), and highly reactive (>0.85), respectively. Four digit numbers placed next to the sequence represent the positions of the nts in the LCV genome. “SL1” and “SL2” denote the right and the left apical stem-loops, respectively, of the Y-shape structure (YSS) in WT LCV RNAs 1 and 2. “S3” denotes the basal, closing stem of the YSS. The stop codon (UAG) encoding P4.8 in RNA2 is labeled “Stop”. Gray and blue nucleotides correspond to nucleotides for which information of BzCN reactivity was unavailable and nucleotides of the primer-binding site, respectively. The engineered 3′ linkers are as indicated.

To determine if either SL1 or SL2 could support viral RNA synthesis or YSS formation, we made a series of mutations targeting both SLs of LCV RNA2, and co-inoculated the *in vitro* produced capped transcripts of each mutant with that of WT LCV RNA1 to tobacco protoplasts. ΔSL1 and ΔSL2 (Fig. 5a) failed to accumulate viral RNA (Fig. 5b,c) suggesting that one SL alone cannot support viral RNA synthesis. SHAPE analysis of the *in vitro* transcripts synthesized from structural cassette plasmids containing ΔSL1 or ΔSL2 revealed that the YSS was lost with the deletion of either SL1 or SL2. However, the deletion of one SL did not eliminate the other SL, although the structure of the latter became slightly altered. For example, in ΔSL1, the basal stem, S3, of the YSS was incorporated into SL2, thereby extending it and forming an additional 1 nt internal loop (compare Fig. 2b,c). In ΔSL2, SL1 was extended due to the incorporation of the nts from the basal stem, S3, of the YSS, and it also formed an additional 5-nt internal loop (compare Fig. 2b,c). Consistent with the previous results, nts in the loop portion of the modified SL1 and SL2 remained reactive to modification (Fig. 2c,d), suggesting that these nts did not interact with other parts of the RNA.

The removal of either SL1 or SL2 could have affected the stability of the transcripts and in turn, directly impacted viral RNA synthesis. To address this possibility, the capped *in vitro* transcripts of LCV RNA2, ΔSL1, or ΔSL2 were each inoculated to protoplasts, and the transcript levels were determined at 3, 12 and 18 hpi by semi-quantitative RT-PCR. The results showed that there was no significant difference in the amount of RT-PCR products generated from the pCM2 (LCV RNA2), ΔSL1, or ΔSL2 inoculations at all time points (Supplementary Fig. S2), suggesting that RNA stability was not affected by the removal of either SLs.

In SLD1-1 and SLD1-2, 6 nts located on either the right arm (SLD1-1) or the left arm (SLD1-2) of the lower stem of SL1 were substituted with the complementary sense nts (Fig. 5a). These mutations disrupted the formation of SL1; none of the *mfold-*predicted models of LCV RNA2 contained the mutated SL1. SLD1-1 and SLD1-2 both failed to synthesize viral RNA (Fig. 5d,e). Unlike SLD1-1 and SLD1-2, SLR1, with a compensatory mutation that restored SL1 (Fig. 5a), synthesized viral RNA to levels comparable to that of the WT (Fig. 5f).

### Exchanging the YSS of LCV RNAs 1 and 2

We next asked whether the YSS of RNA1 could also participate in viral RNA synthesis. Here, we swapped the cDNAs corresponding to the 98-nt of RNA1 (R1) and RNA2 (R2), resulting in chimeras pR1-3′R2 and pR2-3′R1 (Fig. 6a). Although the 98-nt of LCV RNA1 (and RNA2) covers all but 9 nts that make up the proximal 5′ end (the left basal stem) of the YSS, exchanging it with its 98-nt counterpart in LCV RNA2 (or RNA1) was predicted by *mfold* to not affect the formation of the YSS in the resulting chimeric RNAs since 7 of the 9 nts in this region are identical between RNAs 1 and 2 (Fig. 2a,b). The capped *in vitro* transcripts of the two chimeras and that of pCM1 (R1), and pCM2 (R2) were inoculated to tobacco protoplasts in various combinations [#1–#5] (Fig. 6a). Viral RNA accumulation of R1-3′R2 (whether inoculation was performed using transcripts of R1-3′R2 alone [#4], transcripts of R1-3′R2 and R2-3′R1 [#3], or transcripts of R1-3′R2 and R2 [#2]) were detected, and the accumulation levels were comparable to those of the wild type (R1 and R2) (Fig. 6b,c; compare #2, #3 and #4 with #5). Accumulation levels of R2-3′R1 (whether inoculation was performed using transcripts of R2-3′R1 and R1 [#1], or transcripts of R2-3′R1 and R1-3′R2 [#3]) were also comparable to those of the wild type (Fig. 6d, compare #1 and #3 with #5). 5′ RACE analysis was used to determine the 5′ terminal sequences in the (−)-RNA progenies of the WT LCV and chimeric RNAs. In all cases, the original/intended nts were retained in the (−)-RNA progenies.

### LUC-R2 replicons support translation activity in the presence of LCV RNA1

**Figure 3:**
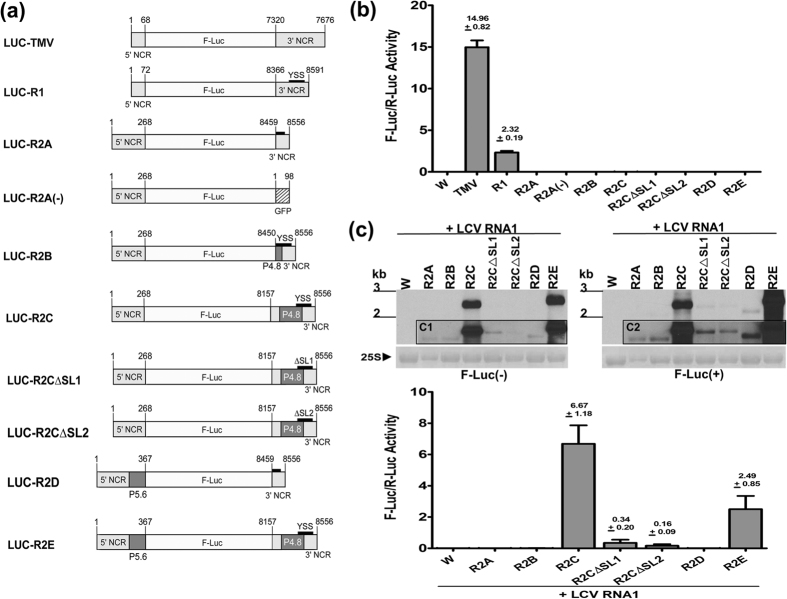
*In vivo* translation assays. **(a**) Reporter constructs with various terminal nt sequences flanking the *firefly* luciferase gene (F-Luc). LUC-TMV: 5′ and 3′ NCRs of TMV30BGFP^49^ RNA; LUC-R1: 5′ and 3′ NCRs of LCV RNA1; LUC-R2A: 5′ and 3′ NCRs of LCV RNA2; LUC-R2A(−), LUC-R2B and LUC-R2C: essentially LUC-R2A except that in LUC-R2A(−), the 3′ NCR is replaced by 98 nts from the GFP gene (striped box), and in LUC-R2B and LUC-2C, the 3′ NCR is extended by adding 9 and 302 nts, respectively, from the immediate upstream region of the LCV RNA 2 3′ NCR; LUC-R2CΔSL1 and LUC-R2CΔSL2: essentially LUC-R2C with stem-loop (SL)1 and SL2, respectively, of the Y-shape structure (YSS) deleted; and LUC-R2D and LUC-R2E: essentially LUC-R2A and LUC-R2C, respectively, except that the 5′ NCR is extended by adding 99 nts from the proximal 5′ end of the P5.6 ORF of LCV RNA2. Complete YSS (black bar labeled “YSS”), partial YSS (unlabeled black bar), and YSS with deleted SL1 (ΔSL1) or SL2 (ΔSL2) are indicated. Light gray and dark gray boxes represent non-coding and coding sequences, respectively. Numbers above the vertical lines in the constructs, except for those at the 3′ flanking region of LUC-R2A(−), are the nt positions in the genomic RNAs of the respective viruses. ((**b,c**) [bottom]) *In vivo* translation assays. Protoplast inoculations were performed using the *in vitro* transcripts of *Renilla* luciferase (R-Luc) and those of F-Luc reporters or water (w; mock inoculation) as indicated; in ((**c**) [bottom]), protoplasts were co-inoculated with the *in vitro* transcripts of cloned LCV RNA1. Means (means <0.009 are not indicated) and standard errors are from relative F-Luc/R-Luc activities determined from triplicate experiments. ((**c**) [top]) Northern analysis of total RNA extracted from protoplasts subjected to the treatments in ((**c**) [bottom]). The polarity of F-Luc RNA being probed is as indicated. Insets (C1 and C2) are 24 hr exposures for each blot. Sizes of RNAs were estimated based on methylene blue-stained RNA standards shown on the left of each blot. The methylene blue-stained 25S rRNA of each sample was included to demonstrate the equal loading of total RNA samples.

In LCV RNA2, ORFs that are located downstream of the first ORF (encoding P5.6) (Fig. 1a) are expressed via a nested set of 3′ co-terminal sgRNAs generated from the (−)-RNA^12^. Because transcripts produced from the LCV RNA2 (R2) series of F-Luc constructs alone do not support translation *in vivo*, this begs the question of how might the cap-dependent translation of P5.6 be achieved. We took the first step towards addressing this question by determining whether or not in the presence of LCV RNA1, RNA transcripts produced from the R2 series of F-Luc constructs could function as replicons to support translation activity. The capped RNA transcripts of constructs: LUC-R2A, -R2B, -R2C, -R2CΔSL1, -R2CΔSL2, -R2D and -R2E were each co-inoculated with that of the internal control R-Luc construct and that of LCV RNA1 into tobacco protoplasts, and the cells were harvested at 72 hpi for analyses. (−)-RNA1 and (+)-RNA1 of LCV were both observed in the Northern blot analyses of all test samples (data not shown). In addition, high levels of (−)-RNA and (+)-RNA synthesis (determined using riboprobes specific to the F-Luc sequence) were observed in the LUC-R2C and -2E samples. In contrast, RNA synthesis in the LUC-R2A, -R2B, -R2CΔSL1, -R2CΔSL2, and -R2D samples were drastically reduced (Fig. 3c). Remarkably, the replication activity of LUC-R2C coincided with a high level of relative luciferase activity (ave. 6.67) (Fig. 3c), while the weak replication of LUC-R2A, -R2B, -R2CΔSL1, -R2CΔSL2, and -R2D coincided with weak or absence of relative luciferase activity (ave. 0.009, 0.01, 0.346, 0.163, and 0.007, respectively) (Fig. 3c). Interestingly, while replication activity in the LUC-R2E sample was as strong as that of the LUC-R2C sample, its relative luciferase activity (ave. 2.49) was reduced (by approx. 2.5 fold) relative to that of the latter (Fig. 3c). These results are discussed in the next section, but a general conclusion is that in the presence of LCV RNA1, the YSS-containing 3′ NCR of LCV RNA2 and its upstream 302 nts support RNA synthesis, and this is accompanied by distinct replicon translation activity.

## Discussion

Successful proliferation within the host cell requires the genome of (+)-RNA viruses to undergo translation and replication. In many cases, these processes are aided by RNA structures located near the termini of the gRNAs. For viruses in the family *Closteroviridae*, a thorough understanding of these processes has been challenging given their large genomes and complex genome expression strategies^9^,^29^. In this study, we have combined SHAPE with biochemical and biological assays to obtain novel insights into the RNA structure and function of a member of this family. SL1 and SL2 are constituents of a higher-order structure (the YSS) that has hitherto not been experimentally determined. Overall, the YSS is much conserved in LCV RNAs 1 and 2, and does not appear to form a pseudoknot as most of the loop nts of SL1 and SL2 can be readily modified by BzCN, suggesting that nts in the loops do not interact with other parts of the RNA (Fig. 2). However, the potentially larger loop in SL2 of RNA2 (reflected in the moderate activity of the A-U and U-A base-pairs) as compared to that in SL2 of RNA1 (Fig. 2a,b) indicates that the two YSSs are not completely identical, suggesting the possibility that their functions might not be entirely the same.

Results from luciferase assays of LUC-R1 suggested that the 5′ and 3′ NCRs of RNA1 are involved in translation. This is not surprising since RNA1 encodes the replicase needed for viral replication. By contrast, similar regions from RNA2 alone, including those upstream of the YSS-containing 3′ NCR, did not support translation in the R2 series of luciferase replicons (Fig. 3b). On this basis, it is likely that in LCV RNA2 alone, the YSS may not function in translation enhancement, unlike for the 3′ cap-independent translational enhancers of viruses that do not have a cap structure at the 5′ end of their genome^30^.

**Figure 4:**
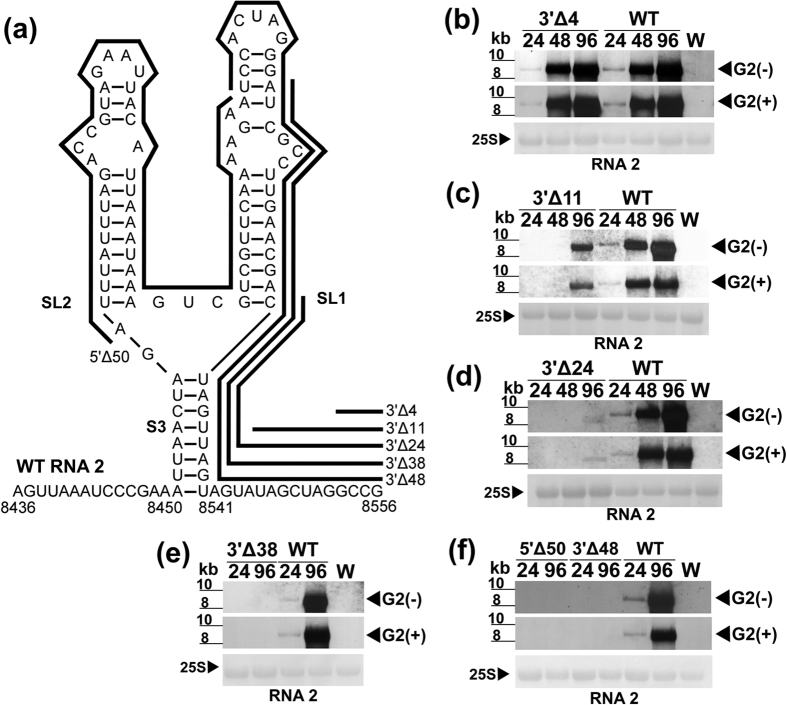
Viral RNA accumulation of LCV RNA2 mutants engineered with deletions in the 3′ terminal region. (**a**) Schematic diagram of the 3′ terminal region of LCV RNA2 (WT RNA2) containing the Y-shaped structure. Deletions were made to the nucleotides (nts) at the proximal 3**′** or 5**′** ends of the 98-nt of RNA2. The number of nts deleted and their proximal locations (marked by bold black lines) are indicated in the names of the mutants: 3′Δ4 (deletion of the last 4 nts), 3′Δ11 (the last 11 nts), 3′Δ24 (the last 24 nts), 3′Δ38 (the last 38 nts), 3′Δ48 (the last 48 nts), and 5′Δ50 (the first 50 nts). Four digit numbers placed next to the sequence represent the nt positions in LCV RNA2. The locations of SL1, SL2 and S3 are as indicated. (**b–f**) Minus- and plus-strand LCV RNA2 accumulation in tobacco protoplasts inoculated with the *in vitro* transcripts of LCV RNA1 and that of each of the LCV RNA2 mutants: 3′Δ4 (**b**), 3′Δ11 (**c**), 3′Δ24 (**d**), 3′Δ38 (**e**), 3′Δ48 (**f**), 5′Δ50 (**f**) or wild type (WT) RNA2 (**b–f**). Total RNA (2 μg each) extracted from the inoculated protoplasts harvested at 24, 48, and 96 hpi (lanes 24, 48 and 96, respectively), and total RNA (2 μg) from water (mock)-inoculated protoplasts harvested at 96 hpi (lane W) were analyzed using DIG-labeled RNA2 negative- or positive-sense specific riboprobe VIII (Fig. 1a). Hybridization signals of minus- and plus-strand genomic RNA2 [G2(−) and G2(+), respectively] are indicated. Estimation of RNA sizes and methylene-blue stained 25s rRNA equal loading controls are as in Fig. 3.

**Figure 5:**
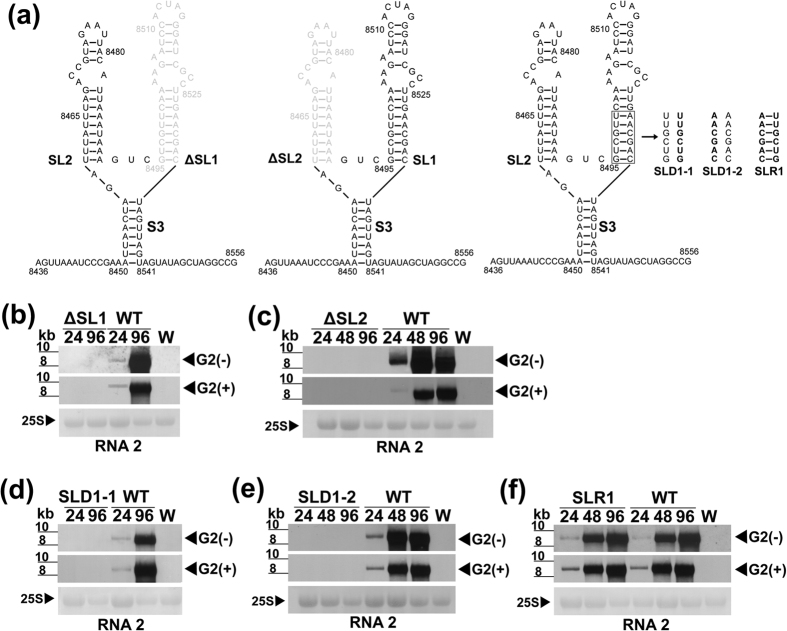
Engineered mutations targeting stem-loops 1 and 2 that form the Y-shape structure in LCV RNA2. (**a**) Schematic diagrams of the secondary structure of SL mutants: ΔSL1 (with SL1 deleted), ΔSL2 (with SL2 deleted), SLD1-1 (with a 6-nucleotide substitution in the right arm of the lower stem of SL1 engineered to disrupt SL1), SLD1-2 (with a 6-nucleotide substitution in the left arm of the lower stem of SL1 engineered to disrupt SL1) and SLR1 (with a compensatory 6-nucleotide substitution in the left arm of the lower stem of SLD1-1 engineered to restore SL1). Light gray letters in ΔSL1 and ΔSL2 represent the nucleotides deleted from SL1 and SL2, respectively. Bold letters in SLD1-1, SLD1-2 and SLR1 represent non-viral nucleotides engineered to substitute the viral nucleotides. The locations of SL1, SL2 and S3 are as indicated. (**b–f**) Tobacco protoplasts were inoculated with the *in vitro* transcripts of wild type LCV RNA1 along with those of LCV RNA2 mutants ΔSL1 (**b**), ΔSL2 (**c**), SLD1-1 (**d**), SLD1-2 (**e**), SLR1 (**f**), or wild type LCV RNA2 (WT). Total RNA (2 μg each) extracted from transcript-inoculated protoplasts 24, 48, and 96 hpi (lanes 24, 48 and 96, respectively), and total RNA (2 μg) extracted from water (mock)-inoculated protoplasts (lane W; harvested at 96 hpi) were analyzed using DIG-labeled negative- or positive-sense specific riboprobe VIII (Fig. 1a). Hybridization signals of minus- and plus-strand genomic RNA2 are indicated as G2(−) and G2(+), respectively. Estimation of RNA sizes and methylene-blue stained 25s rRNA equal loading controls are as in Fig. 3.

**Figure 6:**
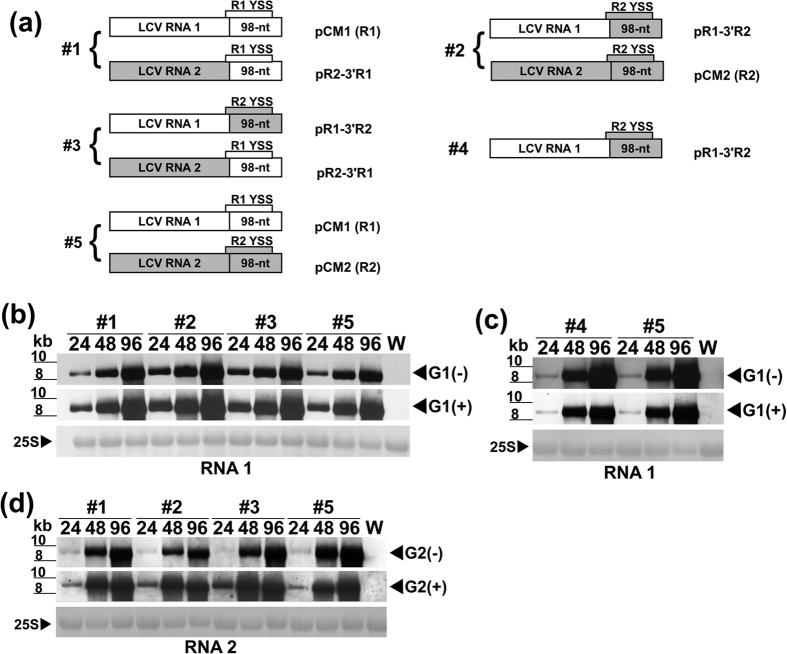
RNA synthesis of LCV RNAs with chimeric YSS. (**a**) Schematic diagrams of the infectious cDNA clones of wild type LCV RNA1 (R1; pCM1), wild type LCV RNA2 (R2; pCM2), mutant LCV RNA1 containing the 98-nt of RNA2 (R1-3′R2), and mutant LCV RNA2 containing the 98-nt of RNA1 (R2-3′R1). The locations of the Y-shape structure (YSS) of R1 (R1 YSS; thin uncolored bar) and R2 (R2 YSS; thin gray bar) relative to the 98-nt of both RNAs are as indicated. Numbers (#1–5) represent different combinations of wild type and/or chimeric mutant *in vitro* transcripts (2 μg each) inoculated into tobacco protoplasts. (**b–d**) Northern blot analyses of RNA accumulation. Northern blots were performed using total RNA (2 μg each) extracted from tobacco protoplasts harvested at 24, 48, and 96 hours (lanes 24, 48, and 96, respectively) following the inoculation of each of the five combinations of *in vitro* transcripts (#1–5); and from water (mock)-inoculated tobacco protoplasts harvested at 96 hours post-inoculation (lane W). Hybridizations were conducted using DIG-labeled negative- or positive-sense specific riboprobes II (**b,c**) and VIII (**d**) (Fig. 1a). Hybridization signals of minus- and plus-strand genomic RNA1 are indicated as G1(−) and G1(+), respectively, and that of minus- and plus-strand genomic RNA2 are indicated as G2(−) and G 2(+), respectively. RNA size estimates and methylene blue-stained 25S rRNA loading controls are as in Fig. 3.

In *Brome mosaic virus* (BMV), TMV and other (+)-RNA viruses^14^,^33^,^34^,^35^,^36^, the last three nts, CC(A/G), in the gRNA(s) are essential for (−)-RNA synthesis. In contrast for LCV, a deletion of as many as 24 nts from the 3′ terminus of RNA2 can be tolerated, although as more nts are deleted, the less efficient viral RNA synthesis becomes (Fig. 4). Based on *mfold* predictions, deleting the nts in 3′Δ11 does not affect the overall structure of the YSS, while deleting those in 3′Δ24 eliminates the right basal stem of the YSS and disrupts its overall structure but leaves both SL1 and SL2 intact (not shown). This suggests that the YSS tolerates a disruption of the basal stem (S3), but cannot tolerate any SL disruptions. Disruptions of either SLs (whether they are large deletions: 3′Δ38, 3′Δ48, and 5′Δ50; targeted deletions: ΔSL1 and ΔSL2; or disruptive substitutions: SLD1-1 and SLD1-2) abolished viral RNA synthesis (Figs 4 and 5). These results suggest that both SLs are needed for viral RNA synthesis. In addition, SHAPE analysis shows that the deletion of one SL eliminates the YSS, but does not disrupt the overall structure of the other SL (Fig. 2). This suggests that SL1 does not serve as a structural support for SL2 and vice versa. Furthermore, deletion of either SL does not appear to affect the overall stability of the RNA (Supplementary Fig. S2). Because the reciprocal exchange of YSS between RNA2 and RNA1 did not affect viral RNA synthesis of the resulting chimeric RNAs (Fig. 6), this suggests that the YSS of RNA1 most likely also participates in viral RNA synthesis. This seems possible given the structural similarity of the YSS of R1 and R2 despite a 22.3% difference in sequence identity^12^. One issue to be addressed in the future is whether the YSS of RNA2 has any role in mediating the messenger activity of RNA1, and if the YSS of RNA1 can confer messenger activity on RNA2.

The experiments in which protoplasts were inoculated with LCV RNA1 and the transcripts of each of the LCV RNA2-based F-Luc constructs were aimed at addressing the lack of *in vivo* translation activity in protoplasts inoculated with the latter alone (Fig. 3b). Prior to these experiments, it was presumed that cap-dependent translation of LCV RNA2 alone was responsible for the expression of P5.6, the first ORF. However, the association of replication and translation for specific R2 replicons co-inoculated with LCV RNA1 (Fig. 3c) demonstrates that the expression strategy is probably more complex than is originally anticipated. This is a novel finding for criniviruses and has led to several new insights. First, replicon RNA synthesis for LUC-R2C and its ΔSL derivatives, i.e. LUC-R2CΔSL1 and LUC-R2CΔSL2, is not dependent on P5.6; even with its coding sequence having been replaced by that of F-Luc (Fig. 3a), RNA synthesis (and luciferase activity) was observed for these replicons (Fig. 3c). For many viruses in the alphavirus-like supergroup, proteins that are non-essential for replication are translated after viral replication has initiated^20^,^37^,^38^,^39^. P5.6 may well be expressed after the (−)-RNA synthesis of LCV RNA2, although the possibility that it is expressed at the same time as, or even before, viral RNA synthesis cannot be completely excluded. It remains possible that the YSS of RNA2 may play a dual role in the LCV infection cycle – that of supporting viral RNA synthesis while also contributing to the translation of P5.6 in the presence of LCV RNA1. However, because P5.6 is not required for replicon RNA synthesis, this lowers the likelihood that the reduction or loss of viral RNA synthesis in the 3′ NCR and/or YSS mutants of LCV RNA2 (Figs 4 and 5) was due to defective P5.6 expression. This means that the role of the YSS in translating P5.6, if at all exists, is possibly independent of that in mediating viral RNA synthesis. Altogether, the above insights lend support to the notion that the YSS of RNA2 contributes to viral RNA synthesis. Targeted mutations engineered in the P5.6 coding sequence of LCV RNA2 will facilitate future work aimed at determining whether it has any role in viral RNA synthesis. Second, RNA synthesis was only efficient for constructs engineered with the YSS-containing 3′ NCR and its 302-nt upstream region (LUC-R2Cand LUC-R2E [Fig. 3c]). However, even with this upstream region present, RNA synthesis was drastically reduced when either of the YSS SLs was deleted (i.e. LUC-R2CΔSL1 and LUC-R2CΔSL2 [Fig. 3c]). The impairment of RNA synthesis for LUC-R2CΔSL1 and LUC-R2CΔSL2 was similar to and consistent with the loss of viral RNA synthesis in the ΔSL1 and ΔSL2 mutants of LCV RNA2 (Fig. 5b,c). Together, this indicates that the YSS serves as an RNA synthesis enhancer in the context of replicon replication, and possibly also in LCV RNA2. Third, since the P4.8 ORF and its potential upstream regulatory region are incorporated in LUC-R2C (and its ΔSL derivatives) and in LUC-R2E, P4.8 is probably produced. However, genome expression analysis suggested that it is translated from a sgRNA transcribed from the (−)-RNA template^12^ i.e. P4.8 is produced downstream of (−)-RNA production; therefore, it is not likely to be involved in (−)-RNA synthesis. Fourth, translation activity corresponded with the replication efficiency of all but one replicon: LUC-R2E, which replicated efficiently but was reduced in translation activity by 2.5 fold relative to LUC-R2C. Thus, it seems that the additional P5.6 coding sequences can have a regulatory effect on translation without interfering with the RNA synthesis enhancer activity of the YSS. The basis for the reduction in translation activity for LUC-R2E is unclear but not unprecedented. Luciferase activity for constructs incorporated with SCV non-coding sequences was reduced when additional nts from the 5′ region of the P26 ORF were incorporated into the 5′ sequence flanking the F-Luc gene^27^.

The simplest explanation for the association between replication and translation involving LCV RNA1 and the R2 replicons is that the YSS facilitates the replicase produced from RNA1^5^,^10^,^40^ to synthesize (−)-RNA, which serves as the template for the production of (+)-RNA from which F-Luc (or P5.6, in the context of LCV RNA2) is expressed. It is possible that the accumulated production of (+)-RNA is needed for the increased translation of a weakly translating message. Our results are also reminiscent of the coupling between replication and translation reported for a number of (+)-RNA viruses with single-component genomes and also for those with segmented genomes^41^,^42^,^43^,^44^,^45^. For example, in *Flock house virus*, a segmented-genome virus also with two gRNAs, cap-dependent translation of a functional CP requires the replication of the encoding RNA (RNA2)^44^. In the case of the bipartite *Red clover necrotic mosaic virus*, cap-independent translation of RNA2, which encodes the movement protein, is linked to its replication in *trans* by the RNA1-encoded replicase^45^. The question remains, how is P5.6 expressed? One possibility is that it can be translated from a sgRNA produced by the (−)-RNA template; a similar strategy has been described for the cap-dependent translation of the coat protein (CP) encoded by the first ORF in RNA2 of *Tobacco rattle virus*^46^. This hypothesis is attractive as it explains the lack of messenger activity in the R2 series of replicons in the absence of LCV RNA1 (Fig. 3b).

A comparison of *mfold-*predicted models generated using the full-length gRNA sequences of criniviruses revealed that structures similar to the YSS are strikingly conserved among other crinivirus genomes (Supplementary Fig. S3). What makes the formation of the YSS possible in these genomes is the conservation of the 3′ NCR in both gRNAs of each crinivirus^8^. An exception to this pattern is found in LIYV, which contains an equivalent of the YSS in the 3′ NCR of RNA1 but not in that of RNA2 largely due to the low (<31%) sequence identity between the two NCRs^31^. On the basis of its position in the crinivirus genomes and the results from our study, it seems likely that the YSS of criniviruses is involved in regulating some common functions associated with viral RNA synthesis. Our data also suggest a biological significance of the YSS in facilitating the similar temporal accumulation of LCV RNAs 1 and 2, where both RNAs were previously found to accumulate in tobacco protoplasts within approximately 12 hpi^5^,^12^. Since LIYV RNA2 lacks the predicted YSS, viral RNA synthesis may require additional enhancements. Indeed, a previous study identified the LIYV RNA1-encoded P34 to be a *trans* enhancer required for the efficient accumulation of LIYV RNA2^10^,^32^. Thus, the structural differences in the 3′ NCR of both LIYV RNAs and requirement of P34 for efficient LIYV RNA2 accumulation may be a basis underpinning the asynchronous RNA replication-accumulation kinetics of LIYV, where the accumulation of RNA2 is delayed by 24 hrs relative to that of RNA1 during the initial stages of infection prior to the production of P34^10^, and this is clearly different from what we see in LCV.

Further studies are needed to delineate the regions/nts of the YSS involved in viral RNA synthesis enhancement, and also to investigate the mechanisms underlying the replication-associated translation of replicons, including issues on whether translation is coupled to de novo replicon replication, the possible involvement of *trans*-activating viral protein(s) or even *trans*-activator structural element(s) present in LCV RNA1^45^,^47^,^48^.

## Methods

### Constructs

Structure cassette plasmids used for SHAPE analysis were engineered with the full-length cDNA sequences of LCV RNA1 (pS-CM1), LCV RNA2 (pS-CM2), LCV RNA2 without SL1 (pS-ΔSL1) and LCV RNA2 without SL2 (pS-ΔSL2). The construction of these plasmids involved using two independent PCR-amplifications. Sequence information of all oligo-primers used for the PCRs is provided in Supplementary Table S1. The first amplification was performed using one of the following oligo-primer pairs and DNA templates: LCV-91-CN/LCV-311-CM and template pCM1, the WT clone of LCV RNA1 (for constructing pS-CM1), LCV-161-CM/ LCV-288-CM and template pCM2, the WT clone of LCV RNA2 (for constructing pS-CM2) or pΔSL2 (for constructing pS-ΔSL2), and LCV-161-CM/LCV-289-CM and template pΔSL1 (for constructing pS-ΔSL1). The resulting products were gel purified and subsequently used for the second amplification in which further modifications were achieved using the reverse oligo-primer LCV-285-CM and one of the following two forward oligo-primers: LCV-91CN (for construction of pS-CM1) and LCV-161-CM (for construction pS-CM2, pΔSL1, and pΔSL2). The amplified products were gel-purified, adenylated, and cloned into the pGEM-T Easy vector. Restriction digestion using HpaI and NdeI (for pS-CM1) or AatII and NgoMIV (for pS-CM2, pΔSL1, and pΔSL2) was performed to release the DNA fragments from the resulting recombinant pGEM-T Easy vectors, and the released DNA fragments were subcloned into similarly digested pCM1 (for making pS-CM1) or pCM2 (for making pS-CM2, pΔSL1, and pΔSL2), resulting in the respective final products.

F-Luc reporter constructs for translation analyses were engineered using pCM1, pCM2 and TMV30BGFP^49^ as templates. Constructs for the determination of viral RNA synthesis were engineered using pCM1 and/or pCM2 as templates^5^. All constructs were engineered using PCR-mediated approaches using the oligo-primers listed in Supplementary Table S1. Details of the making of individual constructs are given in Supplementary Note S1.

All PCR-amplifications were performed using Herculase II fusion DNA polymerase with high fidelity proofreading capability (Agilent Technologies), and all cloned products derived from PCR-amplification were sequenced in both directions.

### SHAPE analysis

5 μg each of pS-CM1, pS-CM2, pS-ΔSL1 or pS-ΔSL2 were linearized by restriction enzyme digestion using NgoMIV. The linearized DNA served as a template for the *in vitro* synthesis of RNA transcripts using the T3 mMessage mMACHINE kit (Life Technologies). 11 μl of dH_2_O containing 2 pmol of the RNA transcript was heated at 95 °C for 2 min, and immediately placed on ice for 1 min. 6 μl of 3.3x RNA folding mix and 1 μl of 100 mM MgCl_2_ was added to the transcript (100 mM HEPES, pH 8.0, 5 mM MgCl_2_, 100 mM NaCl in the final volume), and the mixture was divided into two 9 μl portions and incubated at 37 ^°^C for 10 min to allow the RNA to renature^50^. The first portion of the renatured transcript was treated with 1 μl of 400 mM benzoyl cyanide (BzCN) (Sigma-Aldrich) (40 mM final concentration) made freshly in DMSO at 37 °C for 15 min. The second portion of the renatured transcript (the control treatment) was treated with 1 μl of DMSO without BzCN under the same conditions. RNA was recovered by ethanol precipitation and resuspended in 10 μl of 0.5x TE (5 mM Tris, 0.5 mM EDTA, pH 8)^50^. Primer extension reactions were according to the methods of McGinnis *et al.*^51^. Briefly, BzCN- and DMSO (control)-treated RNA was subjected to primer extension by SuperScript III^®^ reverse transcriptase (Invitrogen) under three consecutive sets of temperature and time conditions: 42 °C for 1 min, 52 °C for 25 min, 65 °C for 5 min using 5′ end-labeled-VIC and -NED 3′-linker primers [5′-GAACCGGACCGAAGCCCGATTTGC-3′, nts complementary to the RT primer binding site of the structure cassette], respectively. Note that the structure cassettes contain a linker region that folds into stable SL structures at the 3′ termini of the transcript upon synthesis. The 3′ linker is designed for the binding of the 5′ end-labeled-VIC and -NED 3′-linker primers and reverse transcriptase (Fig. 2)^52^,^53^. Two separate sequencing reactions, each containing 2 pmol of *in vitro* transcripts produced from the respective structural plasmids, pS-CM1, pS-CM2, pS-3′ΔSL1, or pS-3′ΔSL2, were performed under the same conditions as with the primer extension reaction, except that 1 μl of 5 mM ddTTP was also included in each reaction. In addition, one of the sequencing reactions contained the VIC 3′-linker primer while the other contained the NED 3′-linker primer. The primer extension and sequencing reactions were quenched and combined in the following manner: the BzCN-treated sample with the sequencing sample containing the NED 3′-linker primer, and the DMSO (control)-treated sample with the sequencing sample containing the VIC 3′-linker primer. Following ethanol precipitation, cDNAs were resuspended in 10 μl of deionized formamide and then resolved by capillary electrophoresis. The electropherogram data of each reaction was processed and analyzed by the Qushape software^52^. The reactivity of nts to BzCN modification was classified as: unreactive (0–0.4), moderately reactive (0.4–0.85), or highly reactive (>0.85)^52^. The processed SHAPE reactivities were incorporated as pseudo free energy constraints in *RNAstructure* (version 5.4)^54^ with slope and intercept value of 2.6 and −0.8, respectively, according to Low and Weeks^55^. In addition, base pairing between nucleotides greater than 600 positions was disallowed^55^.

### Luciferase reporter assays

F-Luc reporter constructs (5 μg) were linearized by restriction enzyme digestion using NgoMIV or KpnI (in the case of LUC-TMV). The linearized DNA then served as a template for the *in vitro* synthesis of capped RNA transcripts using the T3 mMessage mMACHINE kit (Life Technologies). The *Renilla* luciferase (R-Luc) construct was linearized by restriction enzyme digestion using SmaI, and capped RNA transcripts were synthesized using the T7 mMessage mMACHINE kit^56^. To perform *in vivo* translation, 3 pmol of the *in vitro* produced capped transcript of each F-Luc reporter construct along with 1 pmol of the *in vitro* synthesized capped R-Luc RNA transcripts were inoculated to half a million *N. tabacum* var. Xanthi protoplasts. In parallel, 1 μl of water was inoculated along with the R-Luc RNA transcripts as a negative control. Additionally, for inoculations that included LCV RNA 1, 2 μg (0.7 pmol) of *in vitro* transcripts synthesized using NgoMIV-linearized pCM1 were used. After 72 hr incubation at 26 °C, cells were harvested and processed using the Dual-Luciferase Reporter Assay System (Promega). Procedures for *in vitro* translation are provided in Supplementary Method S1. Luciferase activity was measured using the Turner Biosystems 20/20n Luminometer (Promega). *In vivo* translation efficiency was determined by the taking the ratio of luminescence produced by F-Luc to that produced by the internal control R-Luc. Both the *in vivo* (Fig. 3b) and *in vitro* (Supplementary Fig. S1) experiments involving the R2 series of luciferase constructs were repeated three times and triplicates of each sample/inoculation were tested in each experiment. All *in vivo* translation experiments involving the R2 series of luciferase constructs and LCV RNA 1 (Fig. 3c) were repeated three times, with duplicate samples for each treatment (see the next section). Scientific graphing and statistical analyses, including two-tailed Student’s t-tests, were performed using the GraphPad Prism 5 software (GraphPad Software, Inc., San Diego, CA).

### *In vitro* transcription, protoplast inoculation, total RNA extraction, and Northern blot analysis

Procedures for the *in vitro* transcription of pCM1, pCM2, the pCM1- and pCM2-engineered mutants followed those previously described^5^, except that restriction digestion for the linearization of p3′Δ11, p3′Δ24, and p3′Δ48 was performed using AgeI.

2 μg of the *in vitro* synthesized transcripts of WT RNAs 1 and 2 (or their engineered derivatives) were each inoculated to *N. tabacum* var. Xanthi protoplasts following the previously described procedure^5^,^57^. To normalize any differences arising from potential uneven handling and inoculation of protoplasts, the following procedure was adopted for inoculations using the WT and the mutants p3′Δ4, p3′Δ11, p3′Δ24, pR1-3′R2, pR2-3′R1, pSLD1-2, pSLR1, and pΔSL2: triplicates of the each combination of *in vitro* transcripts were individually inoculated to 0.5 × 10^6^ protoplasts, and the protoplasts from all three inoculations were combined into a 100 × 20 mm petri dish and incubated at 26 °C. 16 hours post-inoculation (hpi), the combined protoplasts were redistributed into three 60 × 20 mm petri dishes (10 ml per dish) and incubated at 26 °C until the first harvest at 24 hpi. The inoculated protoplasts were also harvested at 48 and 96 hpi. A similar approach was adopted for the *in vivo* translation experiments involving the R2 series of luciferase constructs and LCV RNA1 (Fig. 3c). Duplicates of each inoculated protoplast sample (10 ml each) were combined at 72 hpi and re-distributed into two 10 ml portions; one portion was processed for measuring F-Luc/R-Luc activity and the other portion was saved for Northern Blot analysis. Total RNA was extracted by the TRIzol^®^ method (Invitrogen, Carlsbad, CA) according to the manufacturer’s instructions. Approximately 2 μg of total RNA from each sample were analyzed by Northern Blot using DIG-labeled riboprobes II and VIII as previously described^12^ (Fig. 1a). Signals of viral RNA accumulation on X-ray films were estimated by densitometry using the Scion Image software (Scion Corp) as previously described^12^, or by using the histogram function in Photoshop Element (Adobe Systems) to compute the intensity value and standard deviation using the mean and pixel values of a region of interest in each signal. DIG-labeled riboprobes specific to (+)- or (−)-sense F-Luc RNA were used to determine RNA synthesis for the LCV RNA2 (R2) series of replicons (Fig. 3c). To generate these riboprobes, a recombinant plasmid, pFLuc, containing the cDNA corresponding to a specific location in the F-Luc coding sequence (not present in that of R-Luc) was first obtained. Specifically, using luciferase reporter construct LUC-R2A(−) as template, a 425 nt region specific to F-Luc was amplified using oligonucleotide primer set LUC-005-JZ and LUC-006-JZ (Supplementary Table S1). The PCR amplified product was gel purified and cloned into the pGEM-T Easy vector, resulting in pFLuc. R2 (−)-sense replicons were detected using the (+)-sense F-Luc DIG-labeled riboprobe, which was generated by linearizing pFLuc with SpeI and *in vitro* transcribed with T7 RNA polymerase (Roche Applied Science). R2 (+)-sense replicons were detected using the (−)-sense F-Luc DIG-labeled riboprobe, which was generated by linearizing pFLuc with NcoI and *in vitro* transcribed with Sp6 RNA polymerase (Roche Applied Science).

### Stability assays for input LCV RNA2 transcripts in tobacco protoplasts

2 μg of the *in vitro* synthesized transcripts of pCM2, pΔSL1 and pΔSL2 were each inoculated to *N. tabacum* var. Xanthi protoplasts following the procedures as described above, including the normalization step, except the inoculated protoplasts were redistributed at 3 hpi and harvested at 3, 12, and 18 hpi. Approximately 5 μg of the TRIzol^®^-extracted total RNA from each sample were subjected to DNase treatment (DNA-free™ Kit; Life Technologies). The treated RNA was run on 1% HEPES denaturing gel stained with ethidium bromide to quantitate and normalize the 18S rRNA for equal amounts of rRNA that will be used for cDNA synthesis. 2.5 μg of treated RNA was used to synthesize the first-strand cDNA using Superscript III reverse transcriptase (Invitrogen) and gene-specific oligo-primer LCV-99-AC (Supplementary Table S1) according to the manufacturer’s instruction. 4 μl of the amplified cDNAs was then used for PCR with 2.5 μl of 10X Taq buffer, 2.5 μl of 25 mM MgCl_2_, 1 μl of 10 mM dNTPs, 0.5 μl of Taq DNA polymerase, and 10 μM of oligo-primers LCV-70-PW and LCV-99-AC (Supplementary Table S1). The following PCR conditions were used: 94 °C for 2 min, followed by different number of cycles (10, 15, 20, 25, and 30 cycles) of 94 °C for 45 sec, 54.6 °C for 45 sec, and 72 °C for 1 min, with a final extension at 72 °C for 10 min. For internal control, the same procedure was used to amplify the 18S rRNA as described above except oligo-primer NtUbiR (Supplementary Table S1) was used for reverse transcription, and primers NtUbiF and NtUbiR (Supplementary Table S1) were used for PCR-amplification.

## Additional Information

**How to cite this article**: Mongkolsiriwattana, C. *et al.* A 3′-end structure in RNA2 of a crinivirus is essential for viral RNA synthesis and contributes to replication-associated translation activity. *Sci. Rep.* **6**, 34482; doi: 10.1038/srep34482 (2016).

## Supplementary Material

Supplementary Information
