# Supplementary material for: A 3′-end structure in RNA2 of a crinivirus is essential for viral RNA synthesis and contributes to replication-associated translation activity
Source: Sci Rep. 2016 Oct 3;6:34482. doi: 10.1038/srep34482 (PMC5046102; doi:10.1038/srep34482)
Supplement: Supplementary Information [file srep34482-s1.pdf]

Supplementary Information

A 3'-end structure in RNA2 of a crinivirus is essential for viral RNA synthesis and contributes to replication-associated translation activity

Chawin Mongkolsiriwattana[1,*], Jaclyn S. Zhou[1,*] & James C. K. Ng[1]

[*]Both authors contributed equally to this work

[1]Department of Plant Pathology and Microbiology, University of California, Riverside, Riverside, California, USA

Corresponding author: J.C.K.N

(email: jamesng@ucr.edu)

Note S1

***In vitro luciferase* assays to determine the role of the YSS in translation.** The luciferase activities of LUC-TMV and LUC-R1 were 141- to 181- (ave. 161) fold and 294- to 374- (ave. 334) fold, respectively, higher than that of LUC-R2A, the reference standard (Supplementary Fig. S1a and S1b). These results were consistent with those of the *in vivo* assays (Fig. 3b), suggesting that the 5' and 3' NCRs of LCV RNA1 (but not those of LCV RNA2) supported significant translation activity. We next compared the luciferase activity of LUC-R2A and LUC-R2A(-). In LUC-R2A(-), the GFP nt sequence replacing the 98-nt in LUC-R2A was predicted by *mfold* to not form any secondary structures resembling the SL of the 98-nt. The luciferase activities of LUC-R2A and LUC-R2A(-) were comparably low and not significantly different from each other (Supplementary Fig. S1b). These results were clearly consistent with those of the *in vivo* assays (Fig. 3b), demonstrating that the 5' and 3' NCRs of LCV RNA2 did not participate in translation. To further rule out that the YSS of LCV RNA2 was involved in translation *in vitro,* another series of luciferase reporter constructs were engineered with different lengths of the 5' and 3' terminal sequences of LCV RNA2 (Supplementary Fig. S1a). Construct LUC-R2A was used as a standard to compare how different lengths of the 5' and 3' end sequences of LCV RNA2 might affect the luciferase activity. The luciferase activities of LUC-R2B and LUC-R2C were not significantly different from that of LUC-R2A (Supplementary Fig. S1a and S1b). Furthermore, the luciferase activity of LUC-R2C(-) (Supplementary Fig. S1a), a modified reporter LUC-R2C in which the 3' terminal sequences of LCV RNA2 were substituted with a sequence of identical length (400 nts) randomly selected from the GFP coding sequence, was as low as, and not significantly different from, that of LUC-R2C (Supplementary Fig. S1c). Taken together, this suggests that the terminal 3' (400 nts) sequence, which contains the YSS of

LCV RNA2, does not support translation *in vitro*. To investigate if additional nts from the 5' end of the first ORF (encoding P5.6) might be required for translation of LCV RNA2 *in vitro*, additional luciferase reporter constructs, LUC-R2D and LUC-R2E, were generated (Supplementary Fig. S1a). The luciferase activities of LUC-R2D and LUC-R2E were still minimal, although they were significantly lower than that of LUC-R2A (Supplementary Fig. S1c). However, because the luciferase activities of LUC-R2D (which contained an incomplete YSS) and LUC-R2E (which contained a complete YSS) were not significantly different from each other, this suggested that there was no correspondence between the reduced luciferase activities and the presence or absence of the YSS. This further demonstrates that the 5' NCR and the YSS-containing 3' terminal region of LCV RNA2 are not involved in translation *in vitro*. To determine whether SL1 or SL2 of the YSS of LCV RNA2 was involved in translation, we deleted SL1 or SL2 in LUC-R2C. The resulting constructs, LUC-R2CΔSL1 and LUC-R2CΔSL2 (Supplementary Fig. S1a), showed no significant changes in luciferase activities compared to LUC-R2C (Supplementary Fig. S1c), suggesting that neither SL1 nor SL2 of LCV RNA2 was involved in *in vitro* translation.

Methods

The construction of pCM1 (LCV RNA1)/pCM2 (LCV RNA2) mutants and luciferase reporter constructs. Mutant pR1-3'R2: To replace the 98-nt of LCV RNA1 with that of LCV RNA2, two independent PCRs were performed. The first reaction was performed using oligonucleotide primers LCV-91-CN and LCV-219-CM (with pCM1 as template), while the second reaction was performed using LCV-220-CM and LCV-164-CM (with pCM2 as template). The PCR amplified products were gel-purified using the QIAquick Gel Extraction Kit

(Qiagen, Valencia, CA), and 50 ng of each gel-purified product were subjected to overlapping extension PCR using LCV-91-CN and LCV-164-CM. The resulting product was gel-purified, adenylated and cloned into the pGEM-T Easy vector (Promega Corp., Madison, WI). The recombinant pGEM-T Easy vector was digested with restriction enzymes HpaI and NdeI (New England Biolabs), and the released DNA fragment was subcloned into similarly digested pCM1, resulting in the mutant construct pR1-3'R2.

Mutant pR2-3'R1: replacement of the 98-nt of LCV RNA2 with that of LCV RNA1 was achieved in the same manner as for the replacement of the 98-nt of LCV RNA1 with that of LCV RNA2 as described above, except that the two independent PCRs were performed by using LCV-161-CM and LCV-221-CM (with pCM2 as template), and LCV-222-CM and LCV-223-CM (with pCM1 as template). The overlapping extension PCR was performed using LCV-161-CM and LCV-223-CM, and restriction digestion of the recombinant pGEMT-T Easy vector and pCM2 was made using AatII and NgoMIV.

Mutant p5'Δ50: the pCM2 mutant with a deletion of 50 nts from the proximal 5'-end of the 98-nt of LCV RNA2, was constructed using pCM2 as template, and essentially in the same manner as for the construction of pR1-3'R2 except that the two independent PCRs were performed using LCV-161-CM and LCV-162-CM, and LCV-163-CM and LCV-164-CM, followed by overlapping extension PCR using LCV-161-CM and LCV-164-CM. Restriction digestion of pCM2 and the recombinant pGEMT-T Easy vector containing the overlapping extension PCR product was made using restriction enzymes AatII and ApaI.

Mutants p3'Δ4, p3'Δ11, p3'Δ24, p3'Δ38, and p3'Δ48: the pCM2 mutants with various deletions engineered in the proximal 3'-end of the 98-nt of LCV RNA2 were constructed by using the forward oligonucleotide primer LCV-161-CM and one of the following reverse

primers: LCV-166-CM (p3'Δ4), LCV-176-CM (p3'Δ11), LCV-175-CM (p3'Δ24), and LCV-188-CM (p3'Δ38), and LCV-167-CM (for constructing p3'Δ48). All PCR amplified products were gel-purified, adenylated, and cloned into the pGEM-T Easy vector. Restriction digestions using AatII and ApaI were performed to release the DNA fragments from the resulting recombinant pGEM-T Easy vectors. The released DNA fragments were subcloned into similarly digested pCM2, resulting in the respective final products.

Mutants pΔSL1, pSLD1-1, pSLD1-2 and pSLR1: the pCM2 mutants with specific mutations engineered in the stem loop (SL1) located within the 98-nt (-23 to -61 nts) upstream of the final nt at the 3' end of RNA2 were constructed using pCM2 as template, and in the same manner as for the construction of pR1-3'R2. The first PCR product was generated using the forward oligonucleotide primer LCV-161-CM and one of the following reverse primers: LCV-189-CM (for constructing pΔSL1), LCV-191-CM (pSLD1-1), and LCV-193-CM (pSLD1-2 and pSLR1). The second PCR product was generated using reverse primer LCV-164-CM and one of the following forward primers: LCV-190-CM (for constructing pΔSL1), LCV-192-CM (pSLD1-1), and LCV-194-CM (pSLD1-2 and pSLR1). Overlapping extension PCR of 50 ng of each of the gel-purified form of the above PCR products was performed using oligonucleotide primers LCV-161-CM and LCV-164-CM. The resulting PCR amplified products were gel-purified, adenylated, and cloned into pGEM-T Easy vector. The recombinant pGEM-T Easy vector containing the intermediate product (generated by the overlapping PCR above) to be used for the construction of p3'SLR1 was used as a template for another PCR to introduce the mutation needed to make this mutant. The first PCR was performed using oligonucleotide primer LCV-161-CM and LCV-191-CM; the second PCR was performed using LCV-164-CM and LCV-192-

CM. Overlapping extension PCR (using LCV-161-CM and LCV-164-CM) and cloning of the gel purified products into pGEM-T Easy vector were as described above.

The recombinant pGEM-T Easy vectors containing the PCR products for constructing Mutants pΔSL1, pSLD1-1, pSLD1-2 and pSLR1 were digested with AatII and ApaI, and the released DNA fragments were subcloned into similarly digested pCM2, resulting in the respective final products.

Mutant pΔSL2: the pCM2 mutant engineered without SL2, the stem loop located within the 3'-terminal region (-65 to -96 nts upstream of the final nt at the 3' end) of RNA2, was constructed using pCM2 as template, and in the same manner as that used for constructing pR1-3'R2. The first PCR product was generated using the forward oligonucleotide primer LCV-161-CM and the reverse primer LCV-286-CM. The second PCR product was generated using the forward primer LCV-287-CM and the reverse primer LCV-164-CM. Overlapping extension PCR of 50 ng of each of the gel-purified form of the above PCR products was performed using oligonucleotide primers LCV-161-CM and LCV-164-CM. The resulting PCR amplified products were gel-purified, adenylated, and cloned into pGEM-T Easy vector. The recombinant pGEM-T Easy vectors containing the PCR products were digested with AatII and ApaI, and the released DNA fragments were subcloned into similarly digested pCM2, resulting in pΔSL2.

Luciferase reporter constructs LUC-R1, LUC-R2A, LUC-R2B, LUC-R2C, LUC-R2CΔSL1, LUC-R2CΔSL2, and LUC-TMV were generated in three steps. Step 1: the luciferase T7 Control DNA (Promega) was used as a template to amplify out the luciferase DNA fragment by PCR using the oligo-primers LUC-001-CM and LUC-002-CM. The resulting PCR amplified product was gel-purified and set aside for the next step. Step 2: the bacteriophage T3 promoter followed by nt sequences corresponding to the 5'-terminal region of LCV RNA1, LCV RNA2 or

TMV RNA (each to be engineered upstream of the luciferase gene) were generated by PCR-mediated modifications. The PCRs were performed using one of the following oligo-primer pairs and DNA templates: LCV-89-CN/LCV-290-CM and pCM1 (for construction of LUC-R1), LCV-101-CM/LCV-293-CM and pCM 2 (for construction of LUC-R2A, LUC-R2B, LUC-R2C, LUC-R2CΔSL1, and LUC-R2CΔSL2), and TMV-001-CM/TMV-002-CM and TMV30BGFP[1] (for construction of LUC-TMV). The resulting products were gel-purified and each product, along with the luciferase DNA fragment (from step 1), were subjected to overlapping PCR using one of the following oligo-primer pairs: LCV-89-CN/LCV-292-CM (for construction of LUC-R1); LCV-101-CM/LCV-296-CM (for construction of LUC-R2A), LCV-101-CM/LCV-304-CM (for construction of LUC-R2B), LCV-101-CM/LCV-302-CM (for construction of LUC-R2C, LUC-R2CΔSL1, and LUC-R2CΔSL2); and TMV-001-CM/TMV-005-CM (for construction of LUC-TMV). Thus, the resulting PCR products contained the bacteriophage T3 promoter followed consecutively by the specific viral sequences and the luciferase gene. Step 3: DNA sequences corresponding to the 3'-terminal region of the LCV and TMV30BGFP genomes (each to be engineered downstream of the luciferase gene) were generated by PCR-mediated modifications. The PCRs were performed using one of the following oligo-primer pairs and DNA templates: LCV-291-CM/ LCV-92-CN and pCM1 (for construction of LUC-R1); LCV-303-CM/ LCV-295-CM and pCM2 (for construction of LUC-R2A), LCV-297-CM/LCV-295-CM and pCM2 (for construction of LUC-R2B), LCV-301-CM/LCV-295-CM and pCM2 (for construction of LUC-R2C, LUC-R2CΔSL1, and LUC-R2CΔSL2); and TMV-003-CM/TMV-004-CM and TMV30BGFP (for construction of LUC-TMV). The resulting PCR products were gel-purified and used for overlapping PCRs with the matching products from step 2 (i.e. overlapping PCR was performed using a PCR product with nucleotide sequences corresponding

to the 5' terminal region in the RNA of one virus and one with nucleotide sequences corresponding to the 3' terminal region in the RNA of the same virus). The overlapping PCRs were performed using one of the following oligo-primer pairs: LCV-89-CN/ LCV-92-CN (for construction of LUC-R1), LCV-101-CM/LCV-295-CM (for construction of LUC-R2A, LUC-R2B, LUC-R2C, LUC-R2CΔSL1, and LUC-R2CΔSL2), and TMV-001-CM/TMV-004-CM (for construction of LUC-TMV). The resulting PCR products were gel-purified, adenylated, and cloned into the pGEM-T Easy vector.

Luciferase reporter constructs LUC-R2D and LUC-R2E: To engineer additional nts from the 5' end of the P5.6 ORF to the 5' NCR sequence of LCV RNA2 flanking the luciferase gene in LUC-R2A and LUC-R2C, two PCRs were performed. The first reaction was performed using oligo-primer pairs LCV-101-CM/LCV-305-CM and the pCM2 template. The second reaction was performed using oligo-primer pairs LCV-306-CM/LCV-295-CM and the LUC-R2A or the LUC-R2C template. The PCR amplified products were gel-purified, and 50 ng of each gel-purified product were combined and subjected to overlapping extension PCR using LCV-101-CM and LCV-295-CM. The resulting products were gel-purified, adenylated and cloned into the pGEM-T Easy vector, resulting in LUC-R2D and LUC-R2E.

Luciferase reporter constructs LUC-R2A(-) and LUC-R2C(-): To substitute the LCV RNA2 sequences located downstream of the luciferase gene (in constructs LUC-R2A and LUC-R2C) with nucleotide sequences of equivalent sizes from the coding region of the GFP gene, two independent PCRs were performed. The first reaction was performed using oligo-primer pairs LCV-101-CM/LCV-307-CM and the LUC-R2A or the LUC-R2C template. The second reaction involved amplifying out two different length fragments (98 nts and 400 nts) of the GFP coding sequence from the TMV30BGFP template by using the forward oligo-primer LCV-308-CM and

one of two reverse oligo-primers: LCV-309-CM [for construction of LUC-R2A(-)] or LCV-310-CM [for LUC-R2C(-)] . The resulting products were gel-purified and 50 ng of each gel-purified product were combined and subjected to overlapping extension PCR using oligo-primer pairs LCV-101-CM/LCV-309-CM [for construction of LUC-R2A(-)] or LCV-101-CM/LCV-310-CM [for construction of LUC-R2C(-)]. The resulting product was gel-purified, adenylated and cloned into the pGEM-T Easy vector, resulting in LUC-R2A(-) and LUC-R2C(-).

All standard molecular procedures were performed according to Sambrook and Russell[2]. All cDNA clones were transformed into *Escherichia coli DH5*α competent cells and grown in Luria-Bertani broth containing ampicillin (100 μg/ml; Sigma-Aldrich, St. Louis, MO). All cloned products derived from PCR-amplification were sequenced in both directions.

***In vitro* translation assays.** FLuc reporter constructs (2 μg) were linearized by restriction enzyme digestion using NgoMIV or KpnI (in the case of LUC-TMV). The linearized DNA then served as a template for the *in vitro* synthesis of RNA transcripts using the T3 mMessage mMACHINE kit (Life Technologies). 0.2 pmol of the *in vitro* produced capped transcript of each FLuc reporter construct was added to a mixture containing wheat germ extract (Promega), unlabeled amino acid mixture, potassium acetate (93 mM final concentration), and $MgCl_2$ (2.1 mM final concentration) in a total volume of 25 μl according to the manufacturer's instructions and Wang et al[3]. The reaction was incubated at 25°C for 1 hr, and 2 μl of the reaction was mixed with 50 μl of Luciferase assay reagent (Promega) right before luciferase measurements were taken. Translation activity was based on the FLuc measurement of each sample relative to that of the LUC-R2A sample.

5'-RACE. 5'-Rapid Amplification of cDNA Ends (RACE) was used to analyze the 5' terminus of the minus-sense progenies. Approximately 5 μg of total RNA sample from protoplasts collected at 96 hpi were subjected to reverse transcription by SuperScript II® reverse transcriptase (Invitrogen) using gene-specific primers LCV-27 and LCV-161 (Table S1 in the supplemental material) for cDNA synthesis specific to LCV RNAs 1 and 2, respectively. The first strand cDNAs were purified using QIAquick PCR purification kit. The resulting purified cDNAs were tailed using 1 μl (15U/μl) terminal deoxynucleotidyl transferase (Invitrogen) with either dCTP (for analyzing the progenies generated from p3'Δ4, p3'Δ11, and p3'Δ24), or dTTP (for analyzing the progenies generated from pCM1, pCM2, pR1-3'R2, pR2-3'R1, and pSLR1). The tailed products were subjected to PCR using oligonucleotide primers AAP(T) and LCV-28 (for amplification of RNA1-based sequences from pCM1 and mutant pR1-3'R2), AAP(T) and LCV-103 (for amplification of the RNA2-based sequences from pCM2 and mutants pR2-3'R1, and pSLR1) , or AAP(C) and LCV-103 (for amplification of the RNA2-based sequences from mutants p3'Δ4, p3'Δ11, and p3'Δ24) (Table S1 in the supplemental material). The amplified products were diluted 1:100 in 1 x TE (10mM Tris, 1mM EDTA, pH 8) and used for another round of PCR using oligonucleotide primers AUAP (Invitrogen) and LCV-29, or AUAP and LCV-180 (Table S1 in the supplemental material) for the amplification of RNA1- and RNA2-based sequences, respectively. The cloning of the PCR products into the pGEM-T Easy vector and the subsequent sequencing steps were as described in Text S1 in the supplemental material.

References

1 Rabindran, S. & Dawson, W. O. Assessment of recombinants that arise from the use of a TMV-based transient expression vector. *Virology* **284**, 182-189 (2001).

2 Sambrook, J. & Russell, D. W. *Molecular Cloning: A Laboratory Manual*. 3rd edn (Cold Spring Harbor Laboratory Press, 2001).

3 Wang, Z., Kraft, J. J., Hui, A. Y. & Miller, W. A. Structural plasticity of Barley yellow dwarf virus-like cap-independent translation elements in four genera of plant viral RNAs. *Virology* **402**, 177-186 (2010).

Figure legends

Fig. S1. *In vitro* translation of luciferase-encoding transcripts. (a) Reporter constructs in which the *firefly* luciferase gene (F-Luc) is flanked by various 5' and 3' terminal nucleotide (nt) sequences: LUC-TMV, 5' (68 nts) and 3' (357 nts) NCRs of TMV30BGFP RNA[1]; LUC-R1, 5' (72 nts) and 3' (226 nts) NCRs of LCV RNA1; LUC-R2A, 5' (268 nts) and 3' (98 nts) NCRs of LCV RNA2; LUC-R2B and LUC-R2C, essentially LUC-R2A except that LUC-R2B contains 9 additional nts (from the proximal 3' end of the P4.8 ORF) immediately upstream of the 3' NCR of LCV RNA2, and LUC-R2C contains an additional 302 nts, which include the entire P4.8 ORF and parts of its upstream intergenic region, immediately upstream of the 3' NCR of LCV RNA2; LUC-R2CΔSL1 and LUC-R2CΔSL2, essentially LUC-R2C except that stem-loop (SL)1 and SL2, respectively, in the YSS of LCV RNA2 have been deleted; LUC-R2D and LUC-R2E, essentially LUC-R2A and LUC-R2C, respectively, except that both contain 99 nts (from the proximal 5' end of the P5.6 ORF of LCV RNA2) immediately downstream of the 5' NCR of LCV RNA2; LUC-R2A(-) and LUC-R2C(-), essentially LUC-R2A and LUC-R2C, respectively, except that the nts downstream of F-Luc have been replaced by GFP sequences (striped boxes) of equivalent sizes. A partial YSS is represented by a black bar without the "YSS" label, while the complete YSS is represented by a black bar with "YSS" labeled directly above the bar. The YSS with deletion of either SL1 or SL2 is labeled "ΔSL1" or "ΔSL2", respectively. The light gray and dark gray boxes represent non-coding and coding sequences, respectively. Numbers above the vertical lines in the constructs, except for those at the 3' flanking region of LUC-R2A(-) and LUC-R2C(-), are the nt positions in the genomic RNAs of the respective viruses. (b-c) Relative luciferase activities from *in vitro* translation of the *in vitro* transcripts of LUC-TMV, LUC-R1, LUC-R2A, LUC-R2A(-), LUC-R2B, LUC-R2C, LUC-R2CΔSL1, LUC-R2CΔSL2 ,

LUC-R2C(-), LUC-R2D and LUC-R2E are as indicated. Luciferase activities were measured and normalized to that of LUC-R2A (defined as 1.0). Error bars indicate standard error calculated from three replicated experiments.

Fig S2. Stability of wild type and mutant (ΔSL1 and ΔSL2) LCV RNA2. Tobacco protoplasts were inoculated with 2 μg of each of the following *in vitro* produced capped transcripts: LCV RNA2 (WT), LCV RNA2 YSS mutant ΔSL1, LCV RNA2 YSS mutant ΔSL2, or with water (dH_2O), and harvested 3, 12 and 18 hpi. Total RNA (5 μg) extracted from each sample was treated with DNase and subjected to reverse transcription (RT) using either LCV-99-CM, an LCV RNA2-specific oligo-primer (LCV RNA 2), or NtUbiR, an 18S rRNA-specific oligo-primer (rRNA) (Supplementary Table S1). The PCR amplification for each sample was performed over several different cycles (indicated at the top of each lane) using oligo-primers LCV-99-CM and LCV-70-PW or NtUbiR and NtUbiF (Supplementary Table S1). For the water (dH_2O) inoculated protoplasts, only those harvested at 18 hpi were used for RT-PCR analysis. dH_2O (-) indicates negative (no template; water) control for RT-PCR.

Fig. S3. 3'-terminal *mfold*-predicted secondary structures of selected criniviruses. *Lettuce infectious yellows virus* (LIYV) RNA1, *Bean yellow disorder virus* (BnYDV) RNAs 1 and 2, *Beet pseudo-yellows virus* (BPYV) RNAs 1 and 2, *Blackberry yellow vein associated virus* (BYVaV) RNAs 1 and 2, *Cucurbit chlorotic yellows virus* (CCYV) RNAs 1 and 2, *Cucurbit yellow stunting disorder virus* (CYSDV) RNAs 1 and 2, *Tomato infectious chlorosis virus* (TICV) RNAs 1 and 2, and *Tomato chlorosis virus* (ToCV) RNAs 1 and 2.

Table S1. Oligonucleotide primers used for the construction of engineered mutants, structure cassette plasmids, luciferase reporter constructs, 5' RACE and stability assays.

Primer	Sequence (5' - 3') and polarity (+ or -)	Description	Purpose
LCV-27	TTGAAACCTGTTCAAGACTGCTAAC (+)	Nucleotides corresponding to LCV RNA1 position 7923-7947	5' RACE
LCV-28	ACGAAGGGGTACACCGAATC (+)	Nucleotides corresponding to LCV RNA1 position 8006-8025	5' RACE
LCV-29	TACAGGAAGACCTGTTACTGTTACA (+)	Nucleotides corresponding to LCV RNA1 position 8076-8100	5' RACE
LCV-70-PW	GTGTCAGGTTCTACGTGTCA (+)	Nucleotides corresponding to LCV RNA2 position 7592-7611	Stability Assay (Semi-quantitative RT-PCR)
LCV-89-CN	GGC**AATTAACCCTCACTAAAG**AAATCAAACTTTCCTTCGTACGAAGAG (+)	A T3 promoter sequence (bold) and nucleotides corresponding to those of LCV RNA1 position 1-28 (underlined)	Construction of pLUC-R1
LCV-91-CN	TCATAGTTAACGTATCTGTATCAATAAAGATGTGAGATG (+)	Nucleotides corresponding to LCV RNA1 position 7584-7622	Construction of pS-CM1 and pR1-3'R2
LCV-92-CN	CTGAAT**GCCGGC**GGCCTAGTTATTCTATTAACTAGTCTC (-)	A NgoMIV restriction site (bold) and nucleotides complementary to those of LCV RNA1 position 8591-8564 (underlined)	Construction of pLUC-R1
LCV-99-AC	TTGAGAAGTCTGCTTGAATGTTACT (-)	Nucleotides complementary to LCV RNA2 position 7950-7926	Stability Assay (Semi-quantitative RT-PCR)
LCV-101-CM	*cccgggGCATGC***AATTAACCCTCACTAAAG**AAATTTCCACGGTTTCCCCGAG (+)	XmaI (italic lower case) and SphI (italic upper case) restriction sites; a T3 promoter (bold) and nucleotides corresponding to LCV RNA2 position 1-23 (underlined)	Construction of luciferase reporter constructs of LCV RNA2[3]
LCV-103-CM	ATGGGTTCTTGAACAGTCACTACAGGTTTG (+)	Nucleotides corresponding to LCV RNA2 position 7894-7923	5' RACE
LCV-161-CM	TGATAAGTTGAGAGTGTCCGATCAGG (+)	Nucleotides corresponding to LCV RNA2 position 7312-7337	Construction of various pCM1 and pCM2 mutants[1], pS-CM2, pS-ΔSL1, and pS-ΔSL2, and for 5' RACE
LCV-162-CM	**CCCTAGTGGA**CTAGTTAATTTCGGGATTTA (-)	Nucleotides complementary to LCV RNA2 position 8518-8509 (bold) and 8458-8439	Construction of p5'Δ50
LCV-163-CM	TTAACTAG**TCCACTAGGGATCGCCTTG** (+)	Nucleotides corresponding to LCV RNA2 position 8451-8458 and 8509-8527 (bold)	Construction of p5'Δ50
LCV-164-CM	GGGTTGAGTGTTGTTCCAGTTTG (-)	Nucleotides complementary to position 514-492 of the pGEM-T Easy backbone of pCM2	Construction of various pCM1 and pCM2 mutants[2]

LCV-166-CM	**GGGCCC**GCCGGC*TAGCTATA* (-)	ApaI (bold) and NgoMIV (underlined) restriction sites, and nucleotides complementary to LCV RNA2 position 8552-8544 (italic)	Construction of p3'Δ4
LCV-167-CM	**GGGCCC**ACCGGT*TCTTTTGAACGACGACT* (-)	ApaI (bold) and AgeI (underlined) restriction sites, and nucleotides complementary to LCV RNA2 position 8508-8491 (italic)	Construction of p3'Δ48
LCV-175-CM	**GGGCCC**ACCGGT*CGTTCAAGGCGATCC* (-)	ApaI (bold) and AgeI (underlined) restriction sites, and nucleotides complementary to LCV RNA2 position 8532-8517 (italic)	Construction of p3'Δ24
LCV-176-CM	**GGGCCC**ACCGGT*ACTACTAACTAGTCG* (-)	ApaI (bold) and AgeI (underlined) restriction sites, and nucleotides complementary to LCV RNA2 position 8545-8530 (italic)	Construction of p3'Δ11
LCV-180-AC	TTACGCGTGTGACTTAATTTGAGAG (+)	Nucleotides corresponding to LCV RNA2 position 8290-8314	5' RACE
LCV-188-CM	**GGGCCC**GCCGGC*CCTAGTGGATTCTTT* (-)	ApaI (bold) and NgoMIV (underlined) restriction sites, and nucleotides complementary to LCV RNA2 position 8518-8503 (italic)	Construction of p3'Δ38
LCV-189-CM	**CTACTAACTA**GACTTTATTTAATGTAATTCTACGG (-)	Nucleotides complementary to LCV RNA2 position 8543-8534 (bold) and 8494-8470	Construction of pΔSL1
LCV-190-CM	AATAAAGTC**TAGTTAGTAGTATAGCTAGGCCG** (+)	Nucleotides corresponding to LCV RNA2 position 8486-8494 and 8534-8556 (bold)	Construction of pΔSL1
LCV-191-CM	*ACTA***CAGCAA**CAAGGCGATCCCTAGT (-)	Nucleotides complementary to LCV RNA2 positions 8537-8534 (italic) and 8527-8512 (underlined); and designed for substituting nucleotides from position 8533-8528 with non-viral nucleotides (bold)	Construction of pSLD1-1 and pSLR1
LCV-192-CM	CTTG**TTGCTG***TAGTTAGTAGTATAGCTAGG* (+)	Nucleotides corresponding to LCV RNA2 positions 8524-8527 (underlined) and 8534-8553 (italic); and designed for substituting nucleotides from position 8528-8533 with non-viral nucleotides (bold)	Construction of pSLD1-1 and pSLR1
LCV-193-CM	*TTTG***TTGCTG**GACTTTATTTAATGTAATT (-)	Nucleotides complementary to LCV RNA2 positions 8504-8501 (italic) and 8494-8476 (underlined); and designed for the substituting nucleotides from position 8500-8495 with non-viral nucleotides (bold)	Construction of pSLD1-2 and pSLR1
LCV-194-CM	AGTC**CAGCAA***CAAAAGAATCCACTAGG* (+)	Nucleotides corresponding to LCV RNA2 positions 8491-8494 (underlined) and 8501-8517 (italic); and designed for substituting nucleotides from position 8495-8500 with non-viral nucleotides (bold)	Construction of pSLD1-2 and pSLR1
LCV-219-CM	**CGGTCTAAATAAAT**TTAGTCAATTTTGAGATTTTAC (-)	Nucleotides complementary to LCV RNA2 position 8472-8459 (bold) and LCV RNA1 position 8491-8470	Construction of pR1-3'R2
LCV-220-CM	AATTGACTAA**ATTTATTTAGACCGTAGAATTAC** (+)	Nucleotides corresponding to LCV RNA2 position 8459-8481 (bold) and LCV RNA1 position 8482-8491	Construction of pR1-3'R2
LCV-221-CM	**CTAATATATTTT**CTAGTTAATTTCGGGATTTAAC (-)	Nucleotides complementary to LCV RNA1 position 8503-8492 (bold) and LCV RNA2 position 8458-8437	Construction of pR2-3'R1

LCV-222-CM	CGAAATTAACTAG**AAAATATATTAGACCGTAG** (+)	Nucleotides corresponding to LCV RNA1 position 8492-8510 (bold) and LCV RNA2 position 8446-8458	Construction of pR2-3'R1
LCV-223-CM	GCAGTGAGCGCAACGCAATTA (-)	Nucleotides complementary to position 289-309 of the pGEM-T Easy backbone of pCM1	Construction of pR2-3'R1
LCV-285-CM	gccggc**GAACCGGACCGAAGCCCGATTTGC**GAGCGGCGAACCG*CTCGAG*CGGCC (-)	NgoMIV restriction site (lower case), RT primer binding site (bold), 3'-linker (underlined), XhoI restriction site (italic underlined) and nucleotides complementary to LCV RNA2 position 8556-8552	Construction of pS-CM1, pS-CM2, pS-ΔSL1, and pS-ΔSL2
LCV-286-CM	**GAACGACGAC**TCTAGTTAATTTCGGGATTTAAC (-)	Nucleotides complementary to LCV RNA2 positions 8501-8492 (bold) and 8459-8437	Construction of pΔSL2
LCV-287-CM	ATTAACTAGA**GTCGTCGTTCAAAAGAATCCAC** (+)	Nucleotides corresponding to LCV RNA2 positions 8450-8459 and 8492-8513 (bold)	Construction of pΔSL2
LCV-288-CM	**ATTTGC**GAGCGGCGAACCG*CTCGAG*CGGCCTAGCTATACTACTAACTAGTCGTTC (-)	RT primer binding site (bold), 3'-linker (underlined), XhoI restriction site (italic underlined) and nucleotides complementary to LCV RNA2 position 8556-8527	Construction of pS-CM2 and pS-ΔSL2
LCV-289-CM	**ATTTGC**GAGCGGCGAACCG*CTCGAG*CGGCCTAGCTATACTACTAACTAgacttta (-)	RT primer binding site (bold), 3'-linker (underlined), XhoI restriction site (italic underlined), nucleotides complementary to LCV RNA2 positions 8556-8534 and 8494-8488 (lower case)	Construction of pS-ΔSL1
LCV-290-CM	**ATGTTTTTGGCGTCTTCCAT**TAAGGAAGCGATGTTGAGGG (-)	Nucleotides complementary to luciferase gene position 20-1 (bold) and LCV RNA1 position 72-53	Construction of pLUC-R1
LCV-291-CM	**GCGGAAAGTCCAAATTGTAA**TTAGTGATTAATTAGAAAAT (+)	Nucleotides corresponding to luciferase gene position 1634-1653 (bold) and LCV RNA1 position 8366-8385	Construction of pLUC-R1
LCV-292-CM	ATTTTCTAATTAATCACTAA**TTACAATTTGGACTTTCCGCCC** (-)	Nucleotides complementary to LCV RNA1 position 8385-8366 and luciferase gene position 1653-1632 (bold)	Construction of pLUC-R1
LCV-293-CM	**ATGTTTTTGGCGTCTTCCAT**GATCGTTGACACCAGTTCAG (-)	Nucleotides complementary to luciferase gene position 20-1 (bold) and LCV RNA2 position 268-249	Construction of luciferase reporter constructs of LCV RNA 2[4]
LCV-295-CM	**GGGCCC**GCCGG*CGGCCTAGCTATACTACTAA* (-)	ApaI (bold) and NgoMIV (underlined) restriction sites, and nucleotides complementary to LCV RNA2 position 8556-8537 (italic)	Construction of luciferase reporter constructs of LCV RNA 2[3]
LCV-296-CM	ATTCTACGGTCTAAATAAAT**TTACAATTTGGACTTTCCGCCC** (-)	Nucleotides complementary to LCV RNA2 position 8478-8459 and luciferase gene position 1653-1632 (bold)	Construction of pLUC-R2A
LCV-297-CM	**GCGGAAAGTCCAAATTGTAA**ATTAACTAGATTTATTTAGACCGTAG (+)	Nucleotides corresponding to luciferase gene position 1634-1653 (bold) and LCV RNA2 position 8450-8475	Construction of pLUC-R2B

LCV-301-CM	**GCGGAAAGTCCAAATTGTAA**TTATAATATAGTTATGTATAA (+)	Nucleotides corresponding to luciferase gene position 1634-1653 (bold) and LCV RNA2 position 8157-8177	Construction of pLUC-R2C, pLUC-R2CΔSL1, and pLUC-R2CΔSL2
LCV-302-CM	TATACATAACTATATTATAA**TTACAATTTGGACTTTCCGCCC** (-)	Nucleotides complementary to LCV RNA2 position 8176-8157 and luciferase gene position 1653-1632 (bold)	Construction of pLUC-R2C, pLUC-R2CΔSL1, and pLUC-R2CΔSL2
LCV-303-CM	**GCGGAAAGTCCAAATTGTAA**ATTTATTTAGACCGTAGAAT (+)	Nucleotides corresponding to luciferase gene position 1634-1653 (bold) and LCV RNA2 position 8459-8478	Construction of pLUC-R2A
LCV-304-CM	GTCTAAATAAATCTAGTTAAT**TTACAATTTGGACTTTCCGCCC** (-)	Nucleotides complementary to LCV RNA2 position 8470-8450 and luciferase gene position 1653-1632 (bold)	Construction of pLUC-R2B
LCV-305-CM	**ATGTTTTTGGCGTCTTCCAT**TTCGTCACCCGCCATCGTGAG (-)	Nucleotides complementary to luciferase gene position 20-1 (bold) and LCV RNA2 position 367-347	Construction of pLUC-R2D and pLUC-R2E
LCV-306-CM	CTCACGATGGCGGGTGACGAA**ATGGAAGACGCCAAAAAC** (+)	Nucleotides complementary to LCV RNA2 position 347-367 and luciferase gene position 1-18 (bold)	Construction of pLUC-R2D and pLUC-R2E
LCV-307-CM	TTGAACACCATAAGAGAA**TTACAATTTGGACTTTCCGCCC** (-)	Nucleotides complementary to TMV30BGFP position 5970-5953 and luciferase gene position 1653-1632 (bold)	Construction of pLUC-R2A(-) and pLUC-R2C(-)
LCV-308-CM	**GCGGAAAGTCCAAATTGTAA**TTCTCTTATGGTGTTCAATG (+)	Nucleotides corresponding to luciferase gene position 1634-1653 (bold) and TMV30BGFP position 5953-5972	Construction of pLUC-R2A(-) and pLUC-R2C(-)
LCV-309-CM	gggccc**GCCGGC**GTTCCTGTACATAACCTTC (-)	ApaI (lower case) and NgOMIV (bold) restriction sites, and nucleotides complementary to TMV30BGFP position 6050-6031 (underlined)	Construction of pLUC-R2A(-)
LCV-310-CM	gggccc**GCCGGC**TGGTAAAAGGACAGGGCC (-)	ApaI (lower case) and NgOMIV (bold) restriction sites, and nucleotides complementary to TMV30BGFP position 6352-6334 (underlined)	Construction of pLUC-R2C(-)
LCV-311-CM	**ATTTGC**GAGCGGCGAACCG*CTCGAG*CGGCCTAGTTATTCTATTAACTAGTCTCTC (-)	RT primer binding site (bold), 3'-linker (underlined), XhoI restriction site (italic underlined) and nucleotides complementary to LCV RNA1 position 8591-8562	Construction of pS-CM1
LUC-001-CM	ATGGAAGACGCCAAAAACATAAAG (+)	Nucleotides corresponding to luciferase gene position 1-24	Construction of luciferase reporter constructs[5]
LUC-002-CM	TTACAATTTGGACTTTCCGCCC (-)	Nucleotides complementary to luciferase gene position 1653-1632	Construction of luciferase reporter constructs[5]
TMV-001-CM	GGC**AATTAACCCTCACTAAAG**TATTTTTACAACAATTACC (+)	A T3 promoter sequence (bold) and nucleotides corresponding to those of TMV30BGFP position 1-20 (underlined)	Construction of pLUC-TMV

TMV-002-CM	**ATGTTTTTGGCGTCTTCCAT**TGTAATTGTAAATAGTAATTG (-)	Nucleotides complementary to luciferase gene position 20-1 (bold) and TMV30BGFP position 68-48	Construction of pLUC-TMV
TMV-003-CM	**GCGGAAAGTCCAAATTGTAA**CTATTGTTGTGAGATTTCC (+)	Nucleotides corresponding to luciferase gene position 1634-1653 (bold) and TMV30BGFP position 7320-7338	Construction of pLUC-TMV
TMV-004-CM	GGTACCTGGGCCGCTACCCGCGGTTAG (-)	Nucleotides complementary to TMV30BGFP position 7682-7656 including a KpnI restriction site (underlined)	Construction of pLUC-TMV
TMV-005-CM	GGAAATCTCACAACAATAG**TTACAATTTGGAC TTTCCGCCC** (-)	Nucleotides complementary to TMV30BGFP position 7338-7320 and luciferase gene position 1653-1632 (bold)	Construction of pLUC-TMV
AAP(C)	GGCCACGCGTCGACTAGTACGGGIIGGGIIGGGIIG (-)	5' RACE Abridged Anchor Primer with a poly G/I tract	5' RACE
AAP(T)	GGCCACGCGTCGACTAGTACAAAAAAAAAAAAAAAA (-)	5' RACE Abridged Anchor Primer with a poly A tract	5' RACE
AUAP	GGCCACGCGTCGACTAGTAC (-)	5' RACE Abridged Universal Amplification Primer	5' RACE
NtUbiF	TGCTTAACACATGCAAGTCGGA (+)	Nucleotides corresponding to 18S rRNA	Stability Assay (Semi-quantitative RT-PCR)
NtUbiR	AGCCGTTTCCAGCTGTTGTTC (-)	Nucleotides complementary to 18S rRNA	Stability Assay (Semi-quantitative RT-PCR)
LUC-005-JZ	TGGATACCGGGAAAACGCTG (+)	Nucleotides complementary to *firefly* luciferase gene of replicons in position 1127-1146	Construction of F-Luc probe
LUC-006-JZ	CACAACTCCTCCGCGCAACT (-)	Nucleotides complementary to *firefly* luciferase gene of replicons in position 1532-1551	Construction of F-Luc probe

[1] pR2-3'R1, p5'Δ50, p3'Δ48, p3'Δ4, p3'Δ11, p3'Δ24, p3'Δ38, pΔSL1, pSLD1-1, pSLD1-2, pSLR1, and pΔSL2.

[2] pR1-3'R2, p5'Δ50, pΔSL1, pSLD1-1, pSLD1-2, pSLR1, and pΔSL2.

[3] pLUC-R2A, pLUC-R2B, pLUC-R2C, pLUC-R2CΔSL1, pLUC-R2CΔSL2, pLUC-R2D, and pLUC-R2E.

[4] pLUC-R2A, pLUC-R2B, pLUC-R2C, pLUC-R2CΔSL1, and pLUC-R2CΔSL2.

[5] pLUC-R1, pLUC-R2A, pLUC-R2B, pLUC-R2C, pLUC-R2CΔSL1, pLUC-R2CΔSL2, and pLUC-TMV.

Figure S1

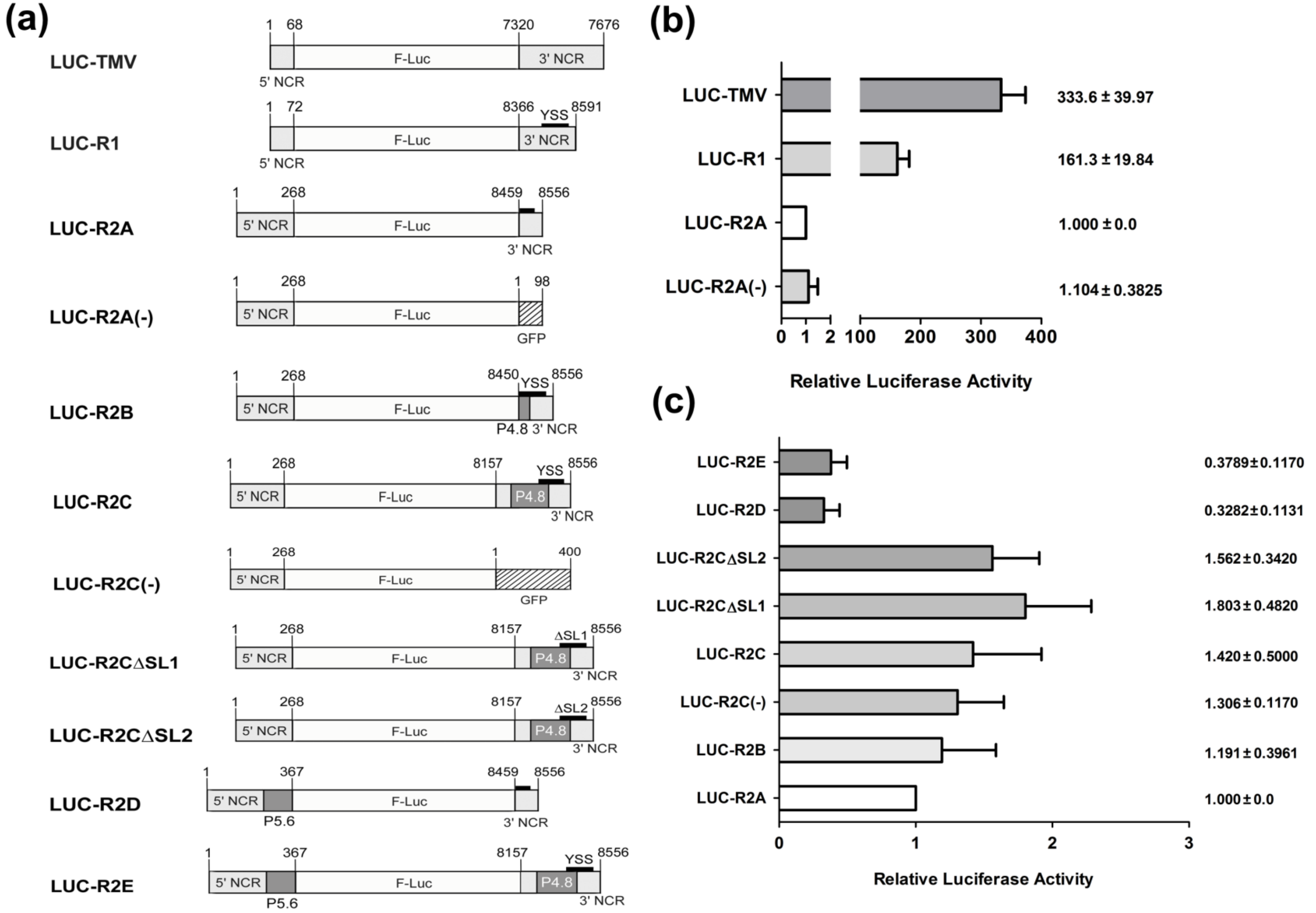
(a)
LUC-TMV
LUC-R1
LUC-R2A
LUC-R2A(-)
LUC-R2B
LUC-R2C
LUC-R2C(-)
LUC-R2C∆SL1
LUC-R2C∆SL2
LUC-R2D
LUC-R2E
5' NCR
F-Luc
3' NCR
YSS
GFP
P4.8
P5.6
∆SL1
∆SL2
(b)
333.6 ± 39.97
161.3 ± 19.84
1.000 ± 0.0
1.104 ± 0.3825
Relative Luciferase Activity
(c)
0.3789 ± 0.1170
0.3282 ± 0.1131
1.562 ± 0.3420
1.803 ± 0.4820
1.420 ± 0.5000
1.306 ± 0.1170
1.191 ± 0.3961
1.000 ± 0.0
Relative Luciferase Activity

Figure S2

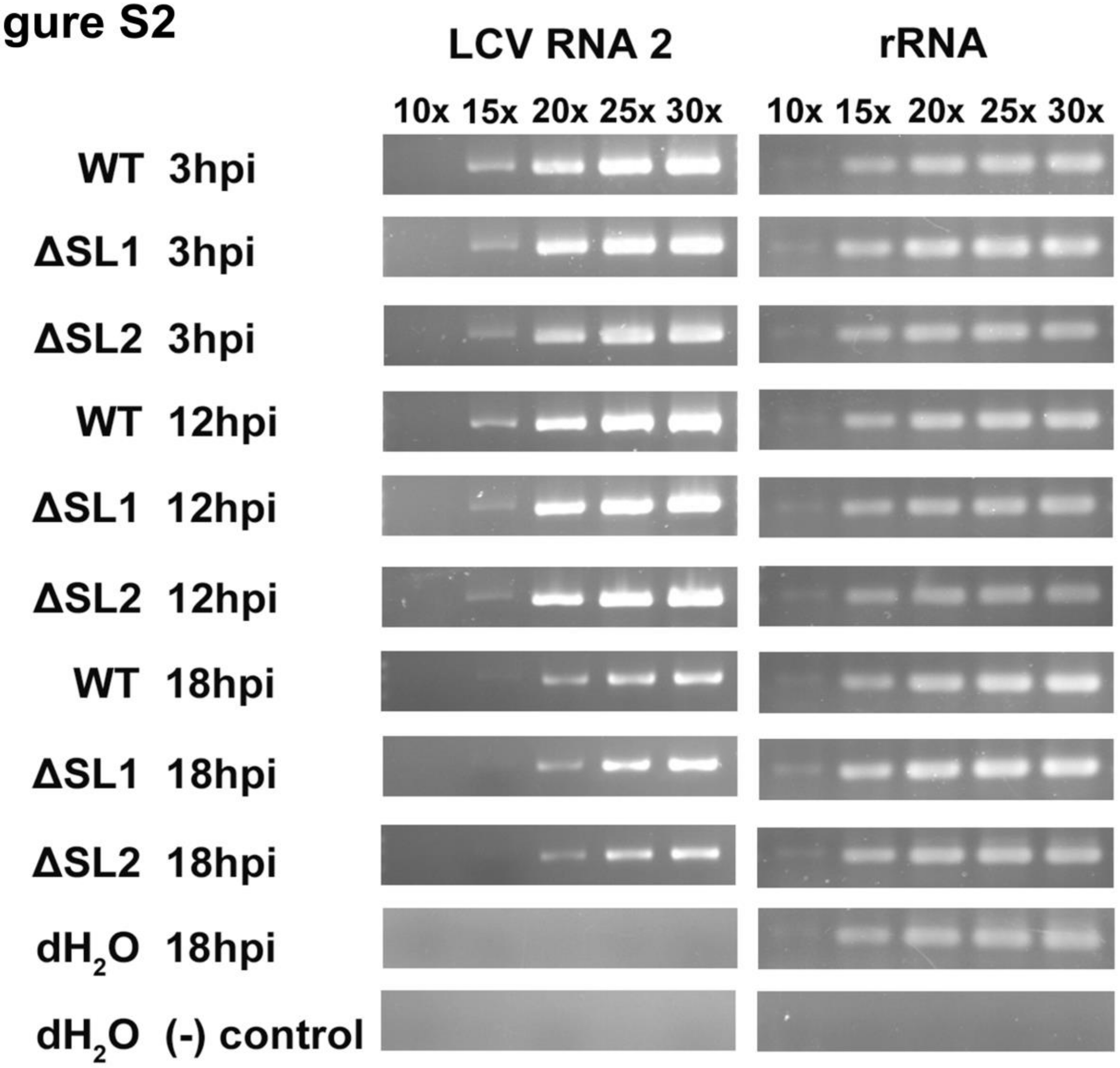
LCV RNA 2
rRNA
10x 15x 20x 25x 30x
10x 15x 20x 25x 30x
WT 3hpi
ΔSL1 3hpi
ΔSL2 3hpi
WT 12hpi
ΔSL1 12hpi
ΔSL2 12hpi
WT 18hpi
ΔSL1 18hpi
ΔSL2 18hpi
dH2O 18hpi
dH2O (-) control

Figure S3

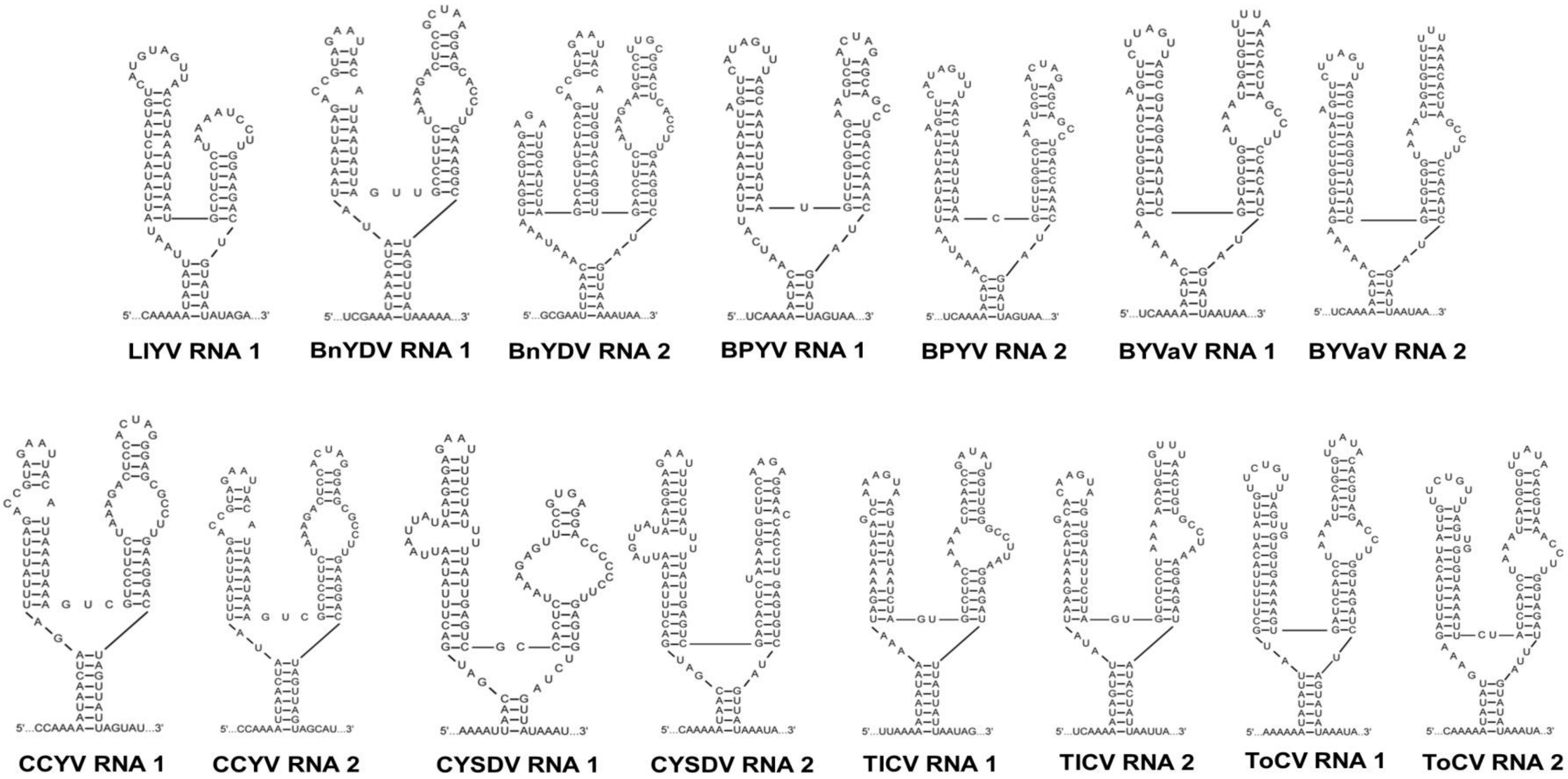
LIYV RNA 1
BnYDV RNA 1
BnYDV RNA 2
BPYV RNA 1
BPYV RNA 2
BYVaV RNA 1
BYVaV RNA 2
CCYV RNA 1
CCYV RNA 2
CYSDV RNA 1
CYSDV RNA 2
TICV RNA 1
TICV RNA 2
ToCV RNA 1
ToCV RNA 2
